# ReaCog, a Minimal Cognitive Controller Based on Recruitment of Reactive Systems

**DOI:** 10.3389/fnbot.2017.00003

**Published:** 2017-01-30

**Authors:** Malte Schilling, Holk Cruse

**Affiliations:** ^1^Center of Excellence Cognitive Interaction Technology, Bielefeld UniversityBielefeld, Germany; ^2^Department of Biological Cybernetics and Theoretical Biology, Bielefeld UniversityBielefeld, Germany

**Keywords:** reactive system, cognitive system; internal model, motor planning, internal simulation, neural networks, attention

## Abstract

It has often been stated that for a neuronal system to become a cognitive one, it has to be large enough. In contrast, we argue that a basic property of a cognitive system, namely the ability to plan ahead, can already be fulfilled by small neuronal systems. As a proof of concept, we propose an artificial neural network, termed reaCog, that, first, is able to deal with a specific domain of behavior (six-legged-walking). Second, we show how a minor expansion of this system enables the system to plan ahead and deploy existing behavioral elements in novel contexts in order to solve current problems. To this end, the system invents new solutions that are not possible for the reactive network. Rather these solutions result from new combinations of given memory elements. This faculty does not rely on a dedicated system being more or less independent of the reactive basis, but results from exploitation of the reactive basis by recruiting the lower-level control structures in a way that motor planning becomes possible as an internal simulation relying on internal representation being grounded in embodied experiences.

## Introduction

Over the last years more and more findings in neuroscience have shown that higher level cognitive capabilities cannot be detached from the functioning of lower level sensorimotor control systems (van Duijn et al., 2006; Barsalou, 2008) which is the core idea of embodied cognition as a field. It is assumed that cognition recruits the underlying sensorimotor systems (Anderson, 2010). Intensively studied examples controlled by such sensorimotor, or reactive, systems are insects. Already a lot is known about their structure and properties of their sensorimotor systems (Menzel et al., 2007; Cruse et al., 2009) which allows to build well performing biologically inspired systems (Pfeifer et al., 2007; Ijspeert, 2014). But it is still unclear if all the crucial properties are understood that are required to form the basis for a cognitive system. Do the known principles allow to leverage the sensorimotor control systems toward cognition?

A basic problem concerns what, after all, is meant by the term “cognition.” Definitions cover various ideas, reaching from Maturana and Varela (1981) “life is cognition” (which would include even bacteria to be cognitive systems), Engel et al. (2013) who note that “cognition is action.” Other authors avoid the problem of a short definition, which almost inevitably includes comparatively simple systems, by listing a collection of phenomena to characterize cognitive systems (e.g., Khlentzos and Schalley, 2007; Menzel et al., 2007). The most important faculties generally agreed as to characterize a cognitive system are attention, awareness, emotion, learning, specific aspects of memory, language as well as thinking, reasoning, planning ahead, decision making, volition, Theory of Mind or even subjective feelings and consciousness (for another list proposed by Langley et al. (2009, see Discussion). In this article, we will not enter this discussion but focus on basic properties discussed by several authors as to be crucial for a cognitive system, namely the ability to invent new behaviors and the ability to plan ahead the latter being required to test the feasibility of the new invention.

Lower level behaviors, often termed reactive or automatic, controlled by “reactive systems,” require procedural elements ensuring survival and allowing for basic behavioral abilities, e.g., locomotion, feeding, object avoidance. The combination of such controllers may also be suited to guide seemingly more complex behaviors (e.g., navigation). These controllers constitute the procedural memory of the system. Exploiting the loop through the world (Brooks, 1989) even a “hard-wired” memory system allows for adaptation to changing environments as will be illustrated in the second section (Reactive Walker). In reactive systems many of these procedures (or “action-perception circuits,” Pulvermüller and Garagnani, 2014) can be active at the same time, but they may also compete amongst each other for controlling the system (Brooks, 1989). Therefore, a crucial ability for each behaving system—including reactive systems—is the ability to select one among different possible actions. This architecture is inspired by earlier authors as Arbib (1998), Brooks (1991b), and Minsky (1986).

Reactive systems, by definition, do not belong to the field of cognition. However, many authors (e.g., Newell, 1994; Anderson, 2010; Glenberg and Gallese, 2012) argue that cognition in all known systems is strongly based on and is intimately connected with a functional reactive system. Even more, as proposed by Barsalou (2008) and others, reactive (or behavior-based) systems having internal states (as introduced in the second section, Reactive Walker) plus being embodied are basic requirements for a system to become a cognitive one. As already noted briefly above, there is indeed strong support showing that neuronal elements forming cognitive properties are tightly intertwined with the reactive system itself and a functional separation is not possible. For example, planning of a movement is interpreted in this view as a mental enactment of the movement (Jeannerod, 2001; Hesslow, 2002). This view is supported as brain regions that formerly were assumed as being highly specialized, for example the motor area, are also activated during language processing or perception (Feldman and Narayanan, 2004; Buccino et al., 2005; Pulvermüller, 2005; Jeannerod, 2006; Pulvermüller and Garagnani, 2014). More generally, Gallese and Lakoff state that “a key aspect of human cognition is. the adaptation of sensory-motor brain mechanisms to serve new roles in reason and language, while retaining their original function as well.” (Gallese and Lakoff, 2005, p. 456). This is supported by behavioral research showing that behavioral and cognitive processes are functionally related insofar as both processes seem to apply the same structuring principles and seem to have access to memory in a structurally similar way (e.g., Jeannerod and Decety, 1995; Cross et al., 2006; Barsalou, 2008; Barsalou et al., 2012).

What distinguishes a reactive system from a cognitive one? A key feature that might be suited for a distinction between reactive, or behavior-based, systems, and cognitive systems is that the former are restricted to apply their procedural memory elements (or internal representations, or internal models) only in the context in which the latter have been acquired (Wilson, 2008). For example, a specific movement (e.g., grasping a specific type of prey) is stored as a (congenital or learned) procedural memory. The content of this memory element may also be considered as a model of that movement, which can—in a reactive system—only be triggered by a specific stimulus, the specific prey. In contrast, cognitive systems are able to modify their behaviors and thereby may come up with solutions for a novel task (Glenberg and Gallese, 2012). A novel task is considered here a task in which, in the current context, none of the existing procedural memory elements can be applied to solve the problem, as none of the available procedures are able to deal with the actual situation or to predict the resulting consequences. Therefore, to approach a cognitive level, one has to search for systems that are creative, i.e., able to alter their procedural memory elements or to compose them in a new way allowing the system to handle such a novel tasks. This characterization agrees with the statement of Limongelli et al. (1995) “cognition is the ability to relate different unconnected pieces of information in new ways and apply the resulting knowledge in an adaptive manner.” Taking a broader view, Anderson (2010), in his massive redeployment hypothesis, states that “neural reuse” is a fundamental principle not only applied in evolutionary time scales but also for solving current problems by a cognitive system. Thus, in this article we will focus on a system that is able to find solutions for novel tasks.

What are the prerequisites to find a solution to a current problem? One way to find new solutions is to apply a search strategy based on simple trial and error. But trial and error is a risky approach and generally quite slow. As an alternative, “internal trial-and-error” could be applied. This means that in addition to the ability to modify the procedures and their composition, such systems are able to anticipate consequences of new actions which enables the agent to decide based on these predictions (Hesslow, 2002). These aspects have already been captured by McFarland and Bösser (1993) who indeed define cognition as the faculty to plan ahead. Planning ahead allows to verify the feasibility of new solutions before execution. Therefore, planning ahead is the second basic property of our system. The ability to predict requires internal models, or internal representations.

Because our system is characterized here as to search for new solutions by exploiting the already existing memories (or internal models) in a flexible way, i.e., not only in a specific context, but in different contextual situations, an organizational scheme is required that allows for compositionality and modulation of specific parameters. In the third section (Motor Planning) we will provide a simple solution for this problem.

Following the view proposed by Barsalou (2008), Glenberg and Gallese (2012) and others, our approach is to start with a non-trivial reactive system that is then equipped with the ability to plan ahead. To this end, we will consider a system with a complex enough body (i.e., having a considerable number of extra degrees of freedom), but an arguably simple controller, which—in order to comply with biological constraints—is based on elements forming an artificial neural network.

Using a system able to control autonomous behavior and using a complex, non-trivial body, we follow a whole-systems approach. We take the embodiment approach literally insofar as our system is constructed in such a way that it is currently used to control a simulated robot in a dynamical simulation environment, but will be transferred to a physical robot in a next step. Thus, we deal with really executable behaviors rather than with more abstract approaches on a dynamical systems level or systems that operate on a symbolic level. Application of such purely high-level approaches may bear the danger that serious problems occurring at a lower level may be overlooked (Brooks, 1991a; Verschure and Althaus, 2003).

Taken together, we focus on a system that allows for the ability to plan ahead (McFarland and Bösser, 1993) relying on intersnal representation (Steels, 2003) that are grounded in embodied experiences (Gallese and Lakoff, 2005). In this way, we follow the proposal of Feynman, who stated that we can only understand a system when we are able to create it (in Hawking, 2001; p. 83). We start with a decentralized, reactive neuronal network controller (Dürr et al., 2004) for a complex hexapod robot which is expanded by a holistic body model represented by a “hard-wired” recurrent neural network (RNN) and used for inverse kinematics (Schilling et al., 2012). Based on a reactive structure the robot allows for walking in an unpredictable environment.

We will further enable the robot to cope with situations for which the reactive system does not offer a solution. In this case, a “cognitive expansion” shall allow the system to search for a new solution to this problem. The search space is not only characterized by the 18°C of freedom (DoF) of the robot, but is expanded by the fact that the controller being embodied heavily depends on the “loop through the world,” i.e., depends on the unpredictable properties of the environment. Further, the complexity of the situation is increased as behavioral elements to be selected show various time dependencies. To cope with such situations, the system first has to search for a behavioral element normally not used in the current context. The search space is large and not continuous. So, gradient descent methods are not applicable. The search for new solutions is based on (i) a somatotopic heuristic, (ii) noise applied to part of the cognitive expansion network as well as (iii) tests for physical feasibility of the solution proposed, first by internal simulation, second by performing the behavior in reality. For internal simulation, we exploit the property of the body model used here, which means that the same model cannot only be used as an inverse model, but also as a predictive model. Therefore, this body model can be used for motor planning applying an internal simulation to test newly selected behavioral elements.

The results show that the cognitive expansion requires only a small number of neurons coupled by a quite simple connectivity. This simple network shows basic properties required for a cognitive system and can be used as a scaffold for later introduction of further properties. In addition, capabilities like showing attention or emotions, might be found as properties emerging from such an architecture as discussed in Cruse and Schilling (2013).

The article is structured in the following way. The second section (Methods and Material) is divided in three parts. In section Background and Previously Developed Models. Reactive Walker—the Walknet (Reactive Walker) the simple control system for a hexapod walker is introduced which is biologically inspired from studies on the walking of insects. In section Motor Planning: from Walknet to reaCog (Motor Planning) the cognitive expansion is presented including an example that illustrates how the basic reactive system is recruited for planning. This will be followed by a more detailed explanation of the control architecture and the experiment setup (section Cognitive Expansion). Simulation results will be presented, on the one hand, for an example scenario (section Results) explaining our approach. On the other hand, a series of simulations shall demonstrate how the approach deals with disturbed walking. While there is no similar robotic architecture which applies behaviors out of context and realizes recruitment as internal simulation, we will present a brief overview on related work and discuss differences and implications (section Related Work). In the Discussion we will analyze the properties of the complete system, discuss them and briefly turn toward the question as to how aspects of higher-level phenomena being listed above may emerge in our system (Discussion and Conclusions).

## Materials and methods

### Background and previously developed models. reactive walker—the walknet

#### Biological model of insect walking

The example we choose as a reactive basis and which will briefly be explained in the following concerns a hexapod (insect-like) walking system (see review Schilling et al., 2013b for details). The task to walk over a non-predictable substrate—possibly cluttered with obstacles of varying size and holes—is by no means a trivial one. The walker has six legs each equipped with three joints. Therefore, the controller has to deal with 18°C of freedom (DoF). As body position in space is defined by only six DoFs (three for position in space, three for orientation) there are 12 DoFs free to be decided upon by the controller which means that the controller has to make these 12 (respectively 18) decisions in a sensible way at any moment of time while dealing with an unpredictable environment. As a first step, the walker is only using tactile sensors situated in the legs (and possibly the antennae Schütz and Dürr, 2011) measuring contact with external objects, and with proprioceptors measuring position, torques and velocities of joints.

The walking system to be described in the following is based on behavioral (and to some extent neurophysiological) studies on insects, in particular stick insects (Schilling et al., 2013b). At first, we briefly describe the essentials of the earlier version, Walknet, and will then introduce expansions.

**Figure 1:**
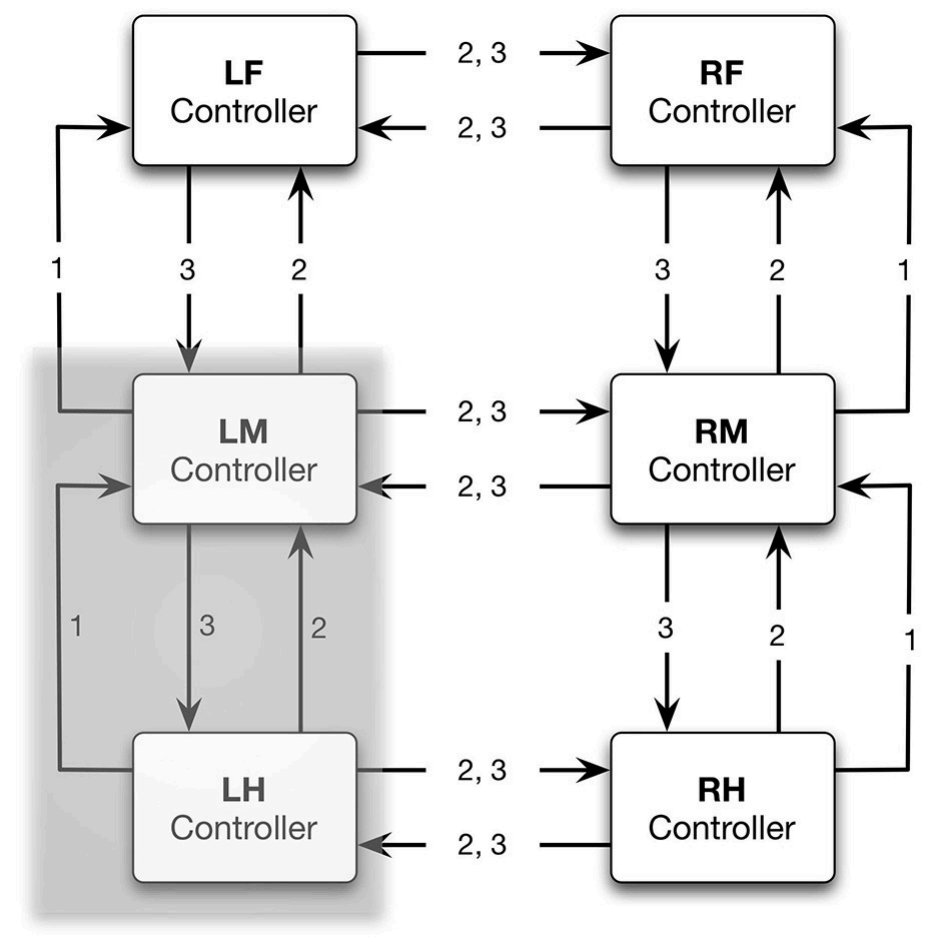
**General architecture of the reactive controller Walknet**. The complete system consists of one controller for each leg (LF/RF left/right front leg, LM/RM left/right middle leg, LH/RH left/right hind leg). Coordination rules (1,2,3) act between neighboring legs, prolonging, or shortening the stance phase. Each leg controller contains several modules, a Swing-net and a Stance-net, to control swing and stance movement, respectively. In Figure 2, the shaded section is depicted in more detail.

**Figure 2:**
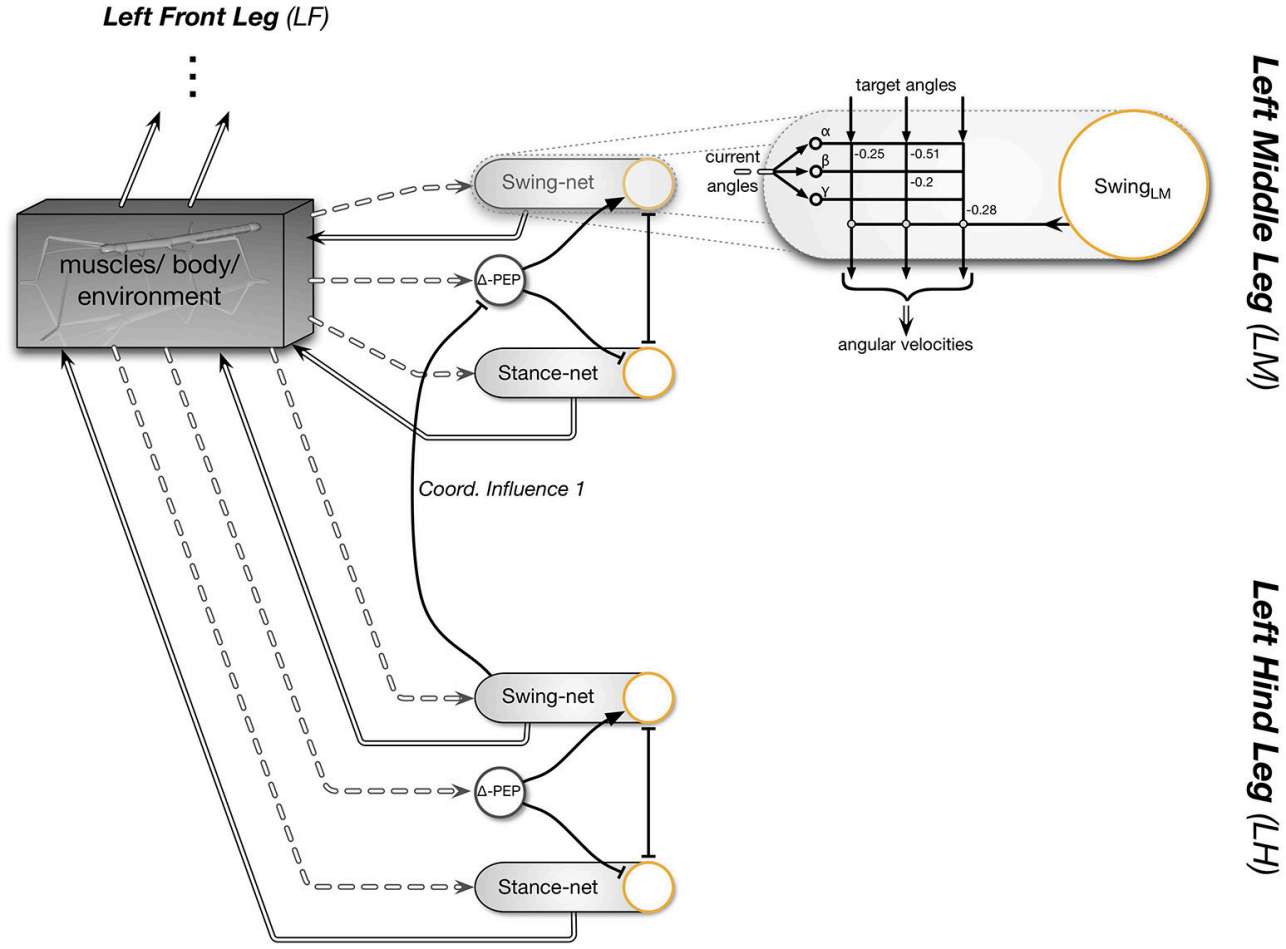
**Interactions between two leg controllers, left middle leg and left hind leg**. This figure details the shaded area of Figure 1. The left side indicates the interaction with the environment mediated through the body. Each leg controller contains several modules: a Swing-net and a Stance-net to control swing and stance movement, respectively, each equipped with a motivation unit (depicted by yellow circles). Connections with an arrow indicate positive (“excitatory”) influences, connections ending with a T-shaped ending indicate negative (“inhibitory”) influences. On the right, one sub-module (Swing-net) is shown in more detail, as it is implemented as a neural network (numbers refer to weights). Target angles serve as an input to the neural network and are stored in the component. Each of the three neural units inside the Swing-net controls the movement of one leg joint. Only one coordination influence is shown in the diagram. In this case, coordination influence 1 (see Figure 1) is acting between the hind and the middle leg. While the hind leg is in swing, the posterior extreme position (PEP) of the anterior leg is shifted backwards and therefore the stance movement is prolonged (Δ-PEP). For further details see Schilling et al. (2013b).

Experiments on the walking stick insect have shown that the neuronal system is organized in a decentralized way (Wendler, 1968; Bässler, 1983; Cruse, 1990). Derived from these results, a model has been proposed in which each leg is attributed a separate controller (Dürr et al., 2004; for a review Schilling et al., 2013b). Figure 1 sketches the approximate anatomical arrangement of the controllers and the numbering of the legs. These single leg controllers are assumed to be situated in the thoracic ganglia (for a review see Bässler and Büschges, 1998). Figure 2 shows details of the controllers as used in Walknet for the left middle leg (LM_leg) and the left hind leg (LH_leg). A single leg controller mainly consists of several movement primitives that reflect the leg movement consisting of two phases. These are the stance movement, during which the leg maintains ground contact and is retracted to propel the body forward, while supporting the weight of the body, and the swing movement where the leg is lifted off the ground and moved in the direction of walking, to touch down at the location where the next stance should begin. The movement primitives controlling stance and swing are realized in the leg controller (Figure 2) as several modules, or procedures, each containing artificial neurons forming a local, in general, recurrent neural network (RNN). These modules might receive direct sensory input and provide output signals that can be used for driving motor elements. The two most important procedural elements in our example are the Swing-net, responsible for controlling a swing movement, and the Stance-net controlling a stance movement (Figure 2, for swing: see Dürr et al., 2004; Schumm and Cruse, 2006, for stance: Schmitz et al., 2008; Schilling et al., 2012). The end positions used during forward walking are stored in the procedures for the swing and stance movement, i.e., the Swing-net and Stance-net respectively (in Figure 2 they are part of the gray rounded boxes called Swing-net and Stance-net. Swing is triggered when the stance-end-position is reached, Stance movement is triggered by ground contact).

**Figure 3:**
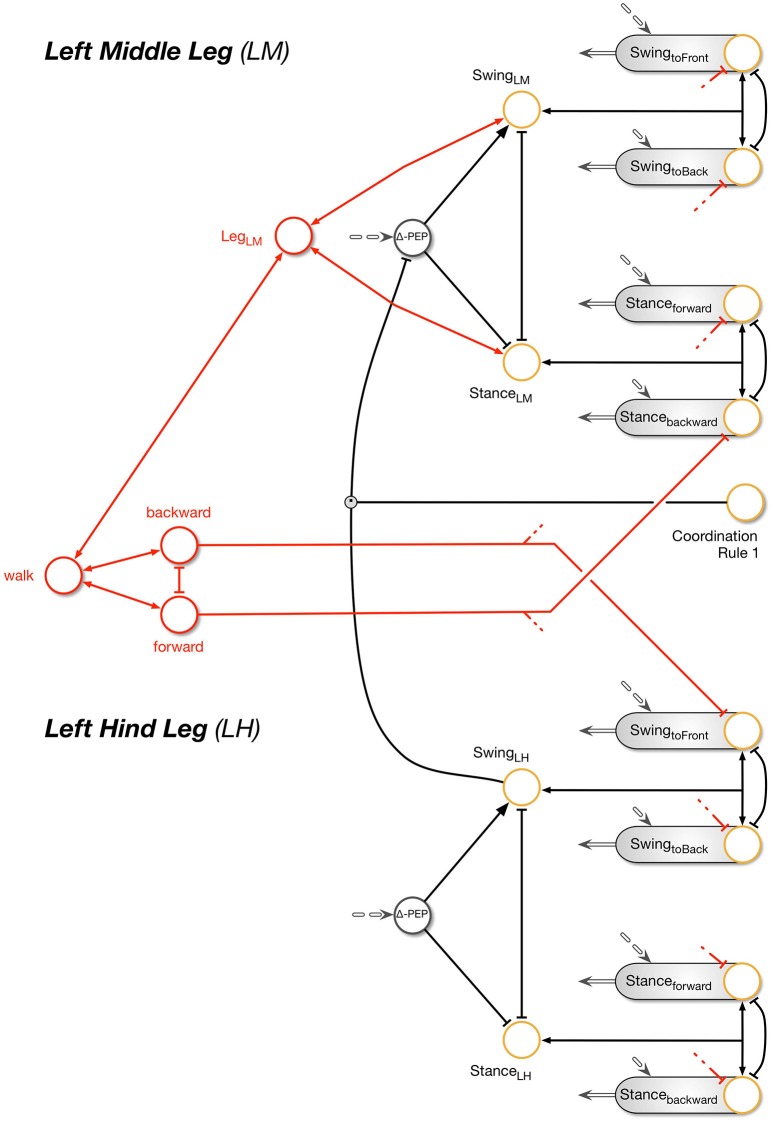
**The extended Walknet**. Compared to the version shown in Figure 2, the ability to walk backwards has been introduced (not all details are shown here.). Each procedural element is equipped with a motivation unit (yellow circle). In addition, the coordination influences (only rule # 1 is depicted) can now be modulated by a motivation unit (yellow circle, coordination Rule # 1). Further motivation units are introduced (red connections and units) being arranged in a heterarchy—again only a fraction of the network is shown (see also Figure 2).

Following Maes (1990) the overall activation of a procedural element is controlled by a motivation unit (represented by yellow circles in the Figures) that gates to what extent the corresponding procedural element contributes to the control of the leg. In the network, these units forming rate coded, non-spiking neurons with leaky integrator, i.e., low pass, dynamics. They have a piecewise linear activation function (from 0 to 1) and control the strength of the output of the corresponding procedure (in a multiplicative way). Here we deal with a very simple motivation unit network that, initially, consists of just two units, the motivation units for the two procedural elements used in forward walking, Swing-net and Stance-net. Each motivation unit is reinforcing itself (not shown in Figure 2) and at the same time inhibiting the other motivation unit, forming a winner-take-all (WTA) net and allowing only one behavior to be active at any given time (Figure 2). Secondly, sensory signals control the behavior selection by influencing the motivation units and thus initiate behavioral transitions. When the leg touches the ground toward the end of a swing movement, the ground contact causes switching to stance movement by activating the motivation unit Stance. Correspondingly, during forward walking, reaching a given posterior position activates the motivation unit Swing. As an extension, we introduced backward walking. In this case, new swing and stance procedures are introduced including their motivation units (Figure 3). Swing_toBack behavior stores the target for the swing movement to the back. As for forward walking, a memory element is required representing the stance end position (for details see Schilling et al. (2013a) and explanation of the Stance movement below).

Furthermore, a leg controller must also take into account the interaction with the other legs. Part of these interactions are mediated directly by the body and through the environment, making explicit computations superfluous (see, e.g., the local positive velocity feedback approach Schmitz et al., 2008). While the physical coupling through the environment is important, it is not sufficient. In addition, the controllers of neighboring legs are coupled via a small number of channels transmitting information concerning the actual state of that leg (e.g., swing, stance) or its position (i.e., values of joint angles). These coordination rules were derived from behavioral experiments on walking sticks (Cruse, 1990). In Figure 1 the channels are numbered 1–3. These coordination rules influence the length of the stance movement by influencing the transition from stance to swing movement, i.e., they change the value of the PEP. In Figure 2 only one connection is shown, influence # 1, which suppresses the start of a swing movement of the anterior leg during the swing movement of the posterior leg (for details see Schilling et al., 2013b).

Beyond the motivation units that are directly controlling a procedural element, there are also motivation units (Figure 3, yellow circles) that are arranged to form some kind of hierarchical structure. Units which belong to the procedural nets controlling the left middle leg show positive connections to a motivation unit termed Leg_LM and this is correspondingly the case for all six legs (only two legs are depicted in Figure 3). These six “leg units” are in turn connected to a unit termed “walk” in Figure 3. This unit serves the function of arousing all units possibly required when the behavior “walk” is activated.

In the case considered here, the motivation unit network, a recurrent neural network, can adopt different stable states, or attractors, forming different overlapping ensembles. For example, all “leg” units and “walk” are activated during backward walking and during forward walking, but only one of the two units termed “forward” and “backward” and only 12 of the 24 end position memories are active in either case. The network is therefore best described as forming a heterarchical structure (for details see Schilling et al., 2013a). Such an “internal state” adopted by the network protects the system to respond to inappropriate sensory input. For instance, as a lower-level example, depending on whether a leg is in swing state, or in stance state, a given sensory input can be treated differently: stimulation of a specific sense organ (not depicted in Figures 2–3, but see Schilling et al., 2013b) leads to a levator reflex when in swing, but not during stance. In other words, the motivation unit network can be considered to act as a top-down attention controller. On higher levels, further internal states could be distinguished, as for example walking, standing still or feeding (for a more detailed discussion on how such a heterarchical network can be structured and learned see Cruse and Schilling, 2010).

The heterarchical structure sketched in Figure 3 comprises a simple realization of neural reuse as proposed in Anderson's massive redeployment hypothesis (Anderson, 2010) as specific procedures are used in different behavioral contexts.

The system as described so far is a slightly expanded version of the earlier Walknet that represents a typical case of an embodied controller (1st order embodiment, c.f. Metzinger, 2006, 2014): Kinematic and dynamic simulations as well as tests on robots have shown that this network can control walking at different velocities, producing different insect gaits including the continuous transitions between the so called wave gait, tetrapod gait and the tripod gait, negotiating curves (Kindermann, 2002), climbing over obstacles (Kindermann, 2002; Dürr et al., 2004), and over very large gaps (Bläsing, 2006), and coping with leg loss (Schilling et al., 2007). Thus, Walknet exhibits a free gait controller where the gaits emerge from a strictly decentralized architecture. Application of this decentralized approach allows for a dramatic simplification of the computation by exploiting the loop through the world (including the own body). For example, trajectories of swing movements are not explicitly given, but result from the cooperation between the Swing-net and the “loop through the world,” i.e., the sensor readings describing the current position of the leg joints. This structure allows for immediate adaptation of swing trajectories to unpredictable disturbances. Similarly, the spatio-temporal patterns of leg movement (“gaits”) are not explicitly specified but result from decentralized local coordination rules and the coupling of the legs via the substrate (see review Schilling et al., 2013b). This network has been tested in dynamic simulation (Schilling et al., 2013a,b) and applied to the robot Hector (Schneider et al., 2011; Paskarbeit et al., 2015). As will be shown in section Motor Planning: from Walknet to reaCog (Motor Planning), this modular structure is a crucial condition to allow recombination of procedural elements as required by a cognitive system.

#### Walknet with a body model

The control of the stance movement is a complex task which requires the coordination of multiple legs and joints. While local embodied approaches can deal with quite complex walking scenarios and disturbances (Schmitz et al., 2008), a purely embodied approach relying on the coupling through the body itself and local leg controllers has shown to become insufficient in other cases (Schilling et al., 2012). For example, stick insects are able to negotiate curves which can be very tight (Dürr, 2005; Dürr and Ebeling, 2005). In the case of curve walking, the different legs are producing quite different movements and are taking over different roles as there is, for example, a differentiation between inner and outer legs. To better cope with such problems, we apply an internal model of the body for the control of the stance movement (Schilling et al., 2012).

Body models are used for three different purposes [for a recent, comprehensive review see Morasso et al. (2015)]. First, inverse models have been applied (e.g., Wolpert and Kawato, 1998) to compute motor commands for given goal positions of an end-effector. The second task concerns the ability to predict the position of the end-effector when motor commands are known but not yet executed (Wolpert and Flanagan, 2001; Webb, 2004). In this case the body model is used as a forward model, for instance to overcome sensory delays. Third, even simple animals as insects use a high number of sensors, for example to measure joint positions or load. In order to exploit this redundancy (e.g., to improve inexact or even missing sensor data), the different sensory inputs have to be fused which requires a body model (Makin et al, 2008). Used for visual perception, the body model, mirroring the observed movement, is strongly related to mirror systems as found in animals (Rizzolatti et al., 1996) and in humans (Rizzolatti, 2005), and might be linked to the understanding of others (Loula et al., 2005).

Whereas, in other approaches usually an individual model has been required for each task and each behavioral element (Wolpert and Kawato, 1998), we use one simple holistic recurrent neural network that can cope with all three tasks. The body model used copes with the at least 18°C of freedom of the insect body (six legs of 3°C of freedom each).

**Figure 4:**
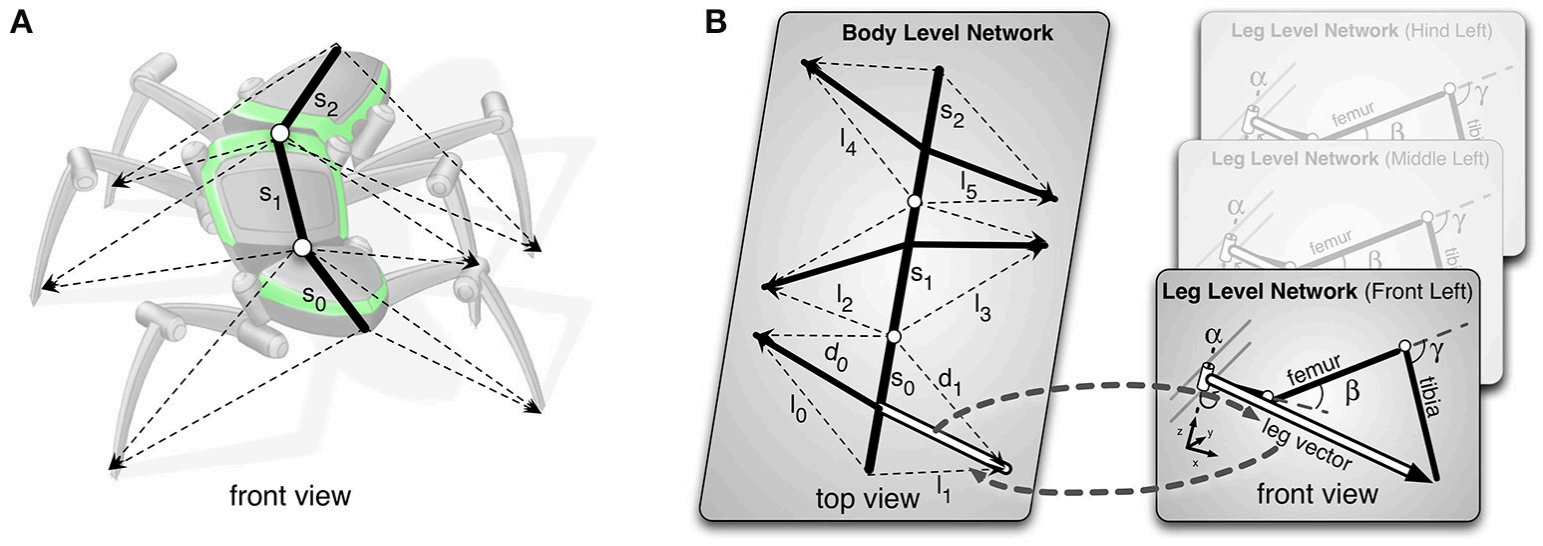
**The body model. (A)** illustrates how the body model (black) represents the body of the robot (gray). **(B)** The Mean of Multiple Computation (MMC) body model for the six-legged walker is divided into two layers. The lower layer contains six networks, each representing one leg (for details see Schilling et al., 2012). The upper layer represents the body and the six legs, which are only represented by bold vectors pointing toward the tip of each leg as shown in **(B)**, left. On this level the leg is described with reference to the respective body segment. Both layers are connected via the shared leg vectors (marked by the double-lined vectors of the left front leg) and are implemented as recurrent neural networks.

The complexity of the six-legged walker is distributed in the body model into interacting submodels (see Figure 4, Schilling and Cruse, 2007). On the lowest level, each leg is represented as a detailed model of all the leg segments and connecting joints [Figure 4B, right; for details see (Schilling, 2011; Schilling et al., 2012)]. These leg models are integrated on a higher level in a model of the central body, where each leg is only represented by a vector pointing from the body segment toward the tip of the leg (Figure 4B, left; for details see Schilling and Cruse, 2012; Schilling et al., 2013a). As this network is based on the principle of pattern completion, any input vector given to the network—may it correspond to the input required for a forward model, an inverse model, or a sensor fusion model—provides an output that, after relaxation, leads to a coherent body state. This means that in any case the kinematics represent a geometrically correct body position. Next, we will explain how this body model can be integrated into the architecture of Walknet.

**Figure 5:**
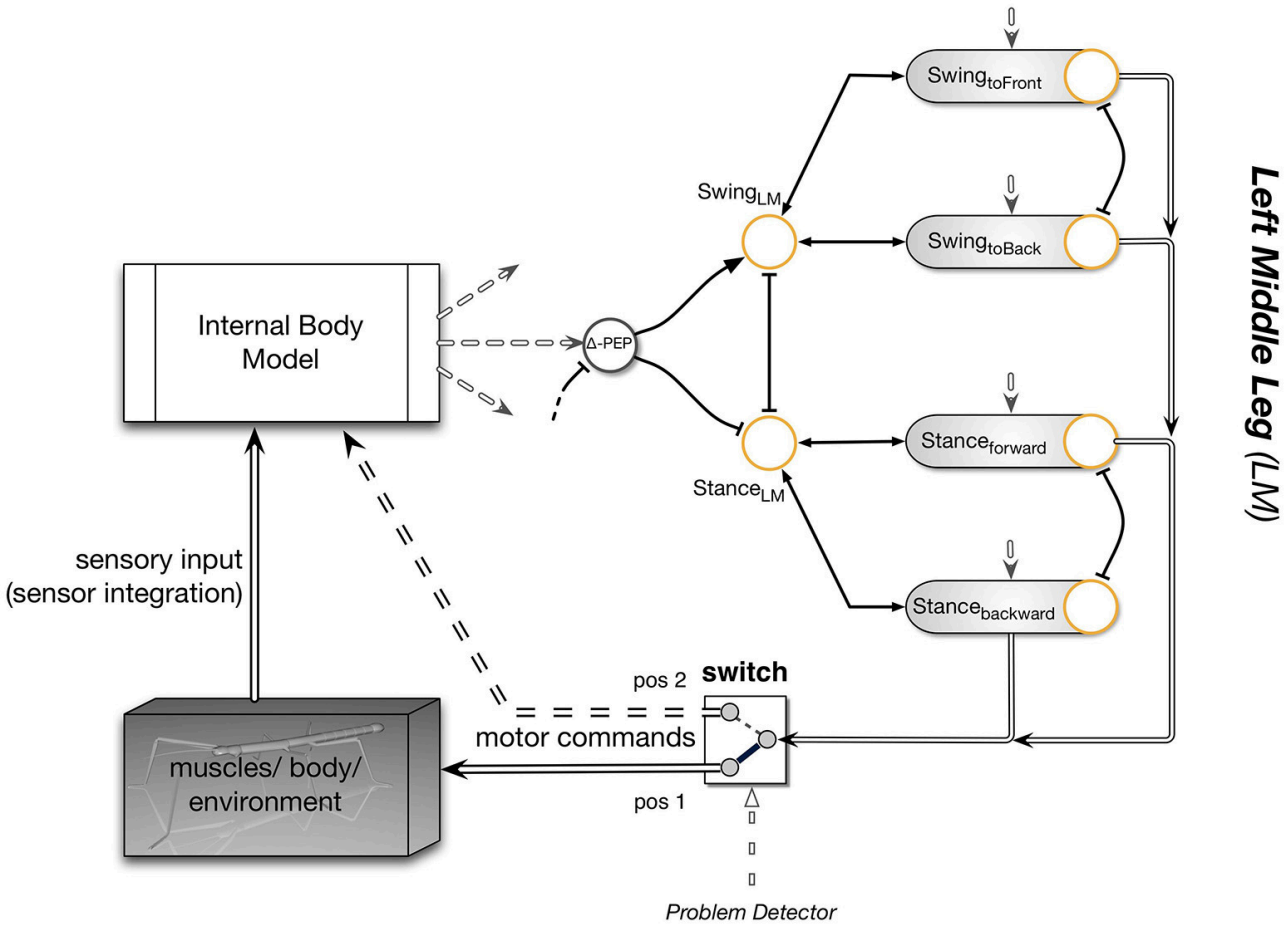
**The first step to reaCog: Walknet expanded by an internal body model**. Only a part of Walknet as shown in Figures 1, 2 is depicted (the left middle leg). During normal behavior, the Internal Body Model (upper left) serves perception. The body provides proprioceptive input (e.g., joint angles from the legs) that is integrated within the body model to form a coherent sensory experience. With the switch in position 1, the network represents a reactive controller. If the system runs into a problem, the switch is flipped from position 1 to position 2 and the motor control (double-lined arrows entering the switch on the right) is routed not to the body anymore, but instead to the body model (dashed double line). This circuit is used for internal simulation and predicts the sensory consequences of the action. The body model is now driven by the motor commands predicting the sensory consequences instead of integrating them. For further explanations see text.

Figure 5 illustrates how the body model is integrated into the network. As depicted in this figure, the internal body model comprises an independent system, which may receive sensory input and/or motor commands. In turn, it provides sensory signals or motor commands to the reactive structure Walknet. The body model can be used for controlling the motor output of the stance behavior in complex walking scenarios. In this case it is part of the reactive controller (in Figure 5 the switch has to take position 1). Using the body model as an inverse model, movement of the legs during stance can easily be controlled by applying the passive motion paradigm (Mussa-Ivaldi et al., 1988). Like a simulated puppet, the internally simulated body is pulled by its head in the direction of desired body movement (Figure 5, sensory input). As a consequence, the stance legs of the puppet follow that movement in an appropriate way and the changes of the simulated joint angles can be used as commands to control the actual joints. Therefore, if such a body model is given, that represents the kinematical constraints of the real body, we obtain an easy solution of the inverse kinematic problem, i.e., for the question how the joints of legs standing on the ground have to be moved in concert to propel the body (for details and application for the control of curve walking see Schilling et al., 2012, 2013a).

In the next section we will introduce a fundamental expansion termed “cognitive expansion.” The complete network, as we will argue, shows how cognitive properties can emerge from a system heavily relying on reactive structures, why we will call this network reaCog.

### Motor planning: from Walknet to reaCog

#### The general idea

To be able to implement the faculty to plan ahead, the neuronal system has to be equipped with a representation of parts of the environment (Schilling and Cruse, 2008; Marques and Holland, 2009). As it has been argued that, as seen from the brain's point of view, the body is the most important part of the environment (Cruse, 2003), a neural representation of the own body is the first step to take. Later, this body model of course has to be extended to include aspects of the environment as are tools extending the body, objects to be handled or an environment to interact with, for example obstacles to be climbed over or to be circumvented.

As mentioned the body model introduced in the previous section can be also used for prediction. Therefore, the body model will be applied to allow the system for being capable of planning ahead through internal simulation.

The basic idea that will be detailed in this section is simple. In short, we will apply the following two-step procedure. If a problem occurs, which means that the ongoing behavior cannot be continued when using only the existing reactive controller, the behavior will be interrupted. The system will then try to come up with new behaviors by recombining the existing procedural elements in a new way, i.e., not envisaged in the current context. A procedural element is characterized by a section of the network that can be controlled by a motivation unit (as shown in Figure 3, red and yellow circles). The properties of the new combination will then be tested by using the internal body model instead of the real body, the former now exploiting its faculty to serve as a forward model. If the new combination turns out to be successful, it will be applied to control the behavior. If not, the system will search for another new combination.

**Figure 6:**
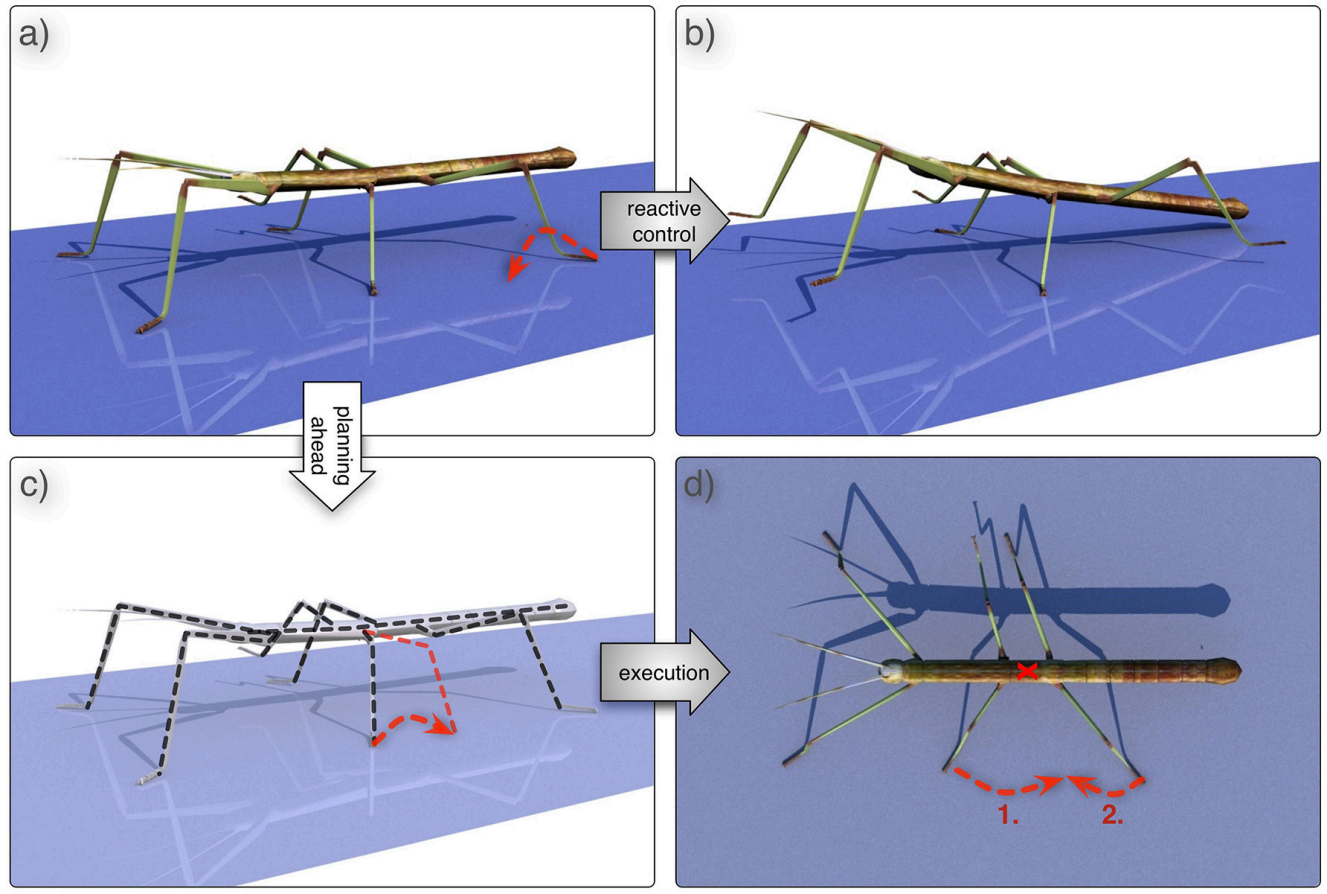
**A problem and a possible solution. (A)** shows a posture in which the animal would fall over when trying to lift the left hind leg (dashed red arrow), because the anterior, middle, leg and the other hind leg are too far to the front. The result is depicted in **(B)**. However, the problem detector detects a problem before the left hind leg is actually lifted, the cognitive system should start searching for a solution through mental simulation **(C)**. The system might come up with the idea to perform a backward swing with its left middle leg and afterwards proceed walking. After successful testing in simulation **(C)**, the plan can be executed in reality **(D)**, i.e., first swinging the middle leg backwards and then swinging the hind leg to the front while continuing normal walking.

For better illustration, we will use the following example: Imagine the case that one—say the left hind leg—has been moved far to the rear and now receives the signal to start a swing movement, i.e., to lift the leg off the ground. If the two neighboring legs—the left middle leg and the other, right, hind leg—accidentally are positioned far to the front, lifting the left hind leg might lead the body falling to the rear (Figures 6A,B).

#### Interruption of behavior

To avoid tumbling over backwards, the system must be able to detect that it is running into trouble. Therefore, one or several systems are necessary that are able to detect that there is a problem. While there are different biologically plausible solutions (e.g., using load sensors as found in the insects), we chose as a simple approach a stability sensor which is activated in case the leg would be lifted,. In the example scenario this detector becomes activated immediately after the motivation unit swing of the hind left leg becomes activated, i.e., before the animal would fall backwards onto the lifted leg.

If a problem has been detected by any detector the system must (i) interrupt the ongoing behavior and (ii) be able to change from the state “perform behavior” to the state “simulate behavior.” To this end, we have introduced a switch as shown in Figure 5. By moving the switch from position 1 to position 2, the output of the leg controller—which is normally (position 1 of the switch) routed to the motor output to influence the body—is now instead routed directly to the body model. Thereby the position of the real body is kept fixed, i.e., the ongoing behavior is interrupted (Hesslow, 2002) is providing a biological account for this decoupling which has also been found in insects (Bläsing and Cruse, 2004), but the internal body model can perform the movements determined by the reactive controller. As in the case of actively moving the body, the output signals of the body model, in particular the angular values describing the position of the leg joints, are given to the reactive procedures. In this way the loop is closed and the system can internally simulate the behavior by moving the body model instead of the real body. Note that modules of the reactive procedures as are Swing-net and Stance-net are still active as is the case in Walknet. 2.2.3 Coming up with a new solution.

This switch given, it appears of course not very interesting to simulate exactly the behavior which has just led to the problem. Instead, it is necessary to test new, currently not available solutions. Therefore, the signal from the problem detectors is not only used to move the switch, but also to start the search for a new solution. To allow for this faculty, reaCog requires a further fundamental expansion.

**Figure 7:**
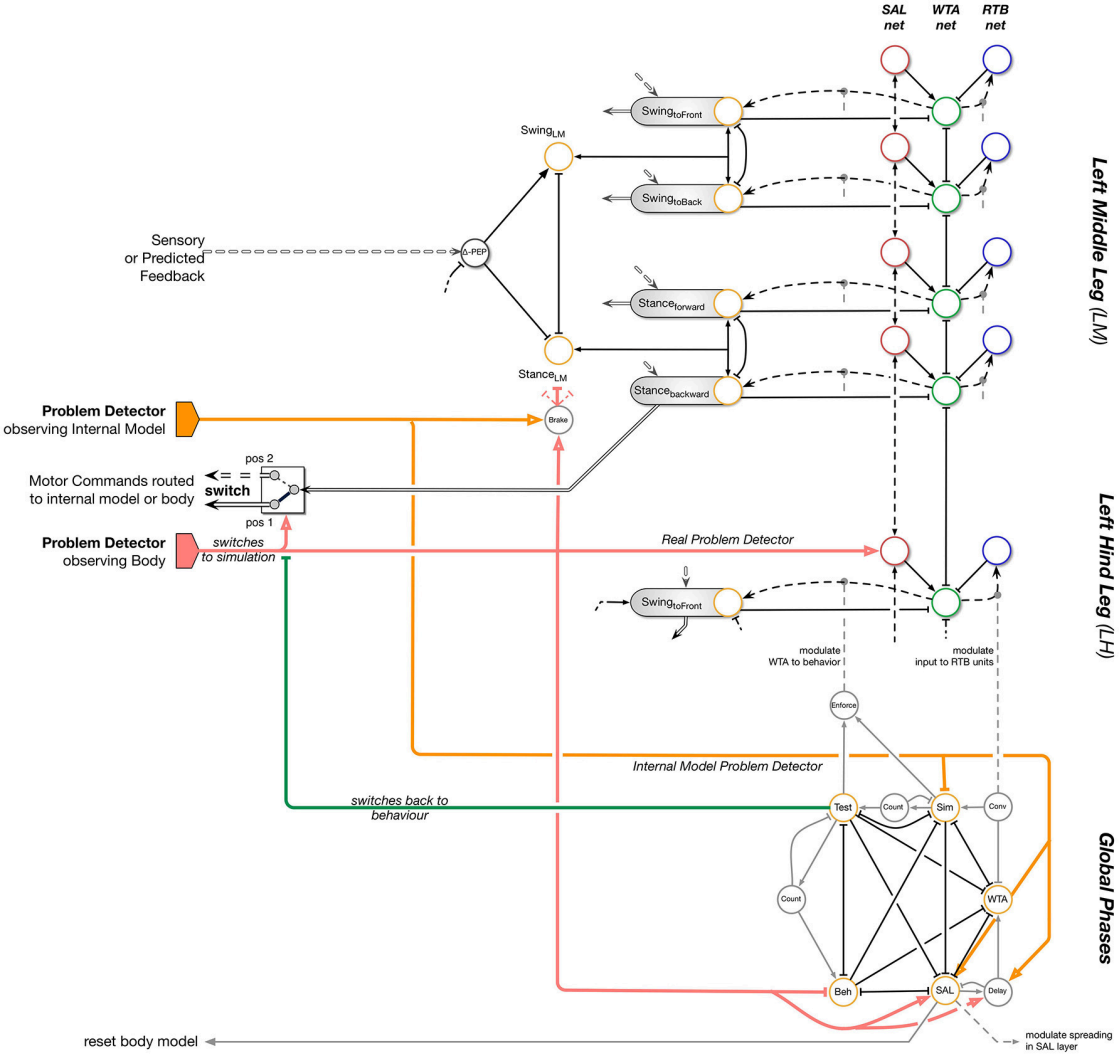
**ReaCog: Walknet plus cognitive expansion**. This figure shows an extension of the Walknet structure presented in Figure 5. The motivation unit structure (yellow, e.g., Swing, Swing_toFront) is replicated on the right side, termed attention system, in three ways. There is a Spreading-Activation-Layer (SAL, red circles), the WTA layer (green circles), and the remember-tested-behavior (RTB units, blue circles) layer. The problem detector (red and yellow, the latter for the internal model) not only activates the switch, but also the spreading activation layer (SAL; red arrows) The activated spreading activation layer units activate their partner units in the WTA network. The winner of the WTA is activating the corresponding motivation unit (dashed black arrows) and the corresponding motor program will be carried out using internal simulation. Note that the connections within the WTA layer are not completely depicted.

The main idea is that for internal simulation a new behavioral element has to be selected. This new behavioral element may be selected also from procedures not belonging to the current context. How is this solved by reaCog? In Figure 7, the upper, left part (i.e., without SAL net, WTA net, and RTB net) shows a simplified version of the network as presented in Figure 5. The expansion depicted at the right side enables the system finding “new solutions” and then testing their qualification to solve the problem. This expansion—that we will call “cognitive expansion” or, as will be motivated in Section Discussion and Conclusions), “attention system”—contains three additional layers, a spreading activation layer (SAL, red circles), a winner-take-all layer (WTA, green circles) and a remember-tested-behavior (RTB, blue circles) layer with identical number of units each. In addition, there is a small network termed Global Phases (lower part of Figure 7).

At the bottom, Global Phases, the structure is illustrated that organizes the temporal sequence of finding a behavior as a solution to a novel problem. Additional units (gray circles) show temporal properties and are used to organize the switching between stages as explained in the text. Units “count” represent a specific time delay.

### Cognitive expansion

In the following we will explain the function of the cognitive expansion as depicted in Figure 7. The goal of the cognitive expansion network is to search for a new procedural element that allows for a solution of the current problem. The first step is to look for behavioral elements existing in the memory, which are, however, not activated in the current context. As will be explained, only such procedural elements can be selected that can be activated by a motivation unit. Second, the possible contribution of this additional memory element will be tested by internal simulation.

How is this done? The units of the SAL (Figure 7, red circles) receive input from morphologically neighboring problem detectors (in Figure 7, one example is depicted by a bold, red circle). Neighboring units are connected by positive weights. In this way, an activation arising from a problem detector is spread over the SAL roughly corresponding to a circular wave starting at the position of the unit excited by the problem detector. Further, there is noise added to the units of the spreading activation layer. The middle layer is representing a winner-take-all network. The units of the WTA layer (Figure 7, green circles) are activated by the corresponding partner units in the SAL layer. In addition, already active behavioral elements, i.e., their active motivation units, are inhibiting their counterparts in the WTA-layer (Figure 7, black solid line with T-shaped end). In this way, currently active behaviors are prevented from being selected for testing in internal simulation. Through the winner-take-all process the units are inhibiting each other in a way that only one unit remains active when the network settles. For the third, the right hand layer, there is again a one-to-one connection to the WTA-layer. These RTB units (Figure 7, blue circles) store which of the WTA units have already been tested in an earlier internal simulation run.

The different procedural elements of Walknet and their motivation units are anatomically arranged in a way that this arrangement coarsely reflects the morphological ordering of the legs (Figure 1, left). Consequently, the motivation units of neighboring legs as well as the partner units of the Spreading Activation layer (SAL) and of the winner-take-all (WTA) layer are neighboring, too, and thus form some kind of somatotopical map. Thus, the problem detector is not only signaling the problem, but in addition also carries some information where the problem occurred. In this way, the search for a new behavior is not purely random, but follows some heuristics,—there is some probability that a solution may be found morphologically near the cause of the problem—which may accelerate the searching process.

**Figure 8:**
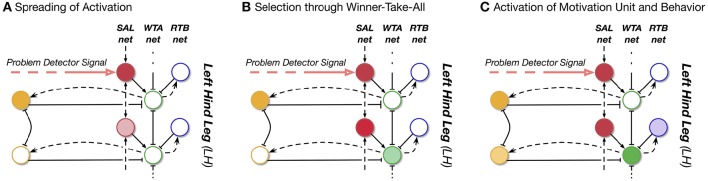
**Illustration of the sequential changes of activation of SAL, WTA, and RTB units**. When a problem occurs, the problem detector, on the one hand stops the execution of current behavior (not shown). On the other hand, it induces activity in the spreading activation layer (SAL, red) which indicates where the problem occurred. The activation is spreading vertically in the SAL. Each SAL unit excites its corresponding WTA unit. Importantly, currently active motivation units (yellow) inhibit the WTA units (green units). The WTA units compete among each other producing one winning unit which in turn activates the corresponding motivation unit and behavior. The units in the RTB layer (blue) represent which behavior has been active once during the simulation process and will inhibit a future activation during the WTA selection process.

What is the functional role of these three additional layers forming an expansion that we will call “cognitive expansion” or, as will be motivated later in the discussion (Section Discussion and Conclusions), “attention system”? Assume that in our example (Figure 5) the problem detector situated in the left hind leg has been activated (Figure 7, bold red arrow, starting at the left). As noted earlier, this signal moves the switch from position 1 to position 2 to route the motor output to the body model instead of the body itself. Thereby the ongoing behavior is interrupted. In addition this signal activates one (or several) neighboring units of the Spreading Activation layer. Figure 8 illustrates the sequential activation of WTA layer, and RTB layer.

The winning WTA unit activates its motivation unit and as a consequence, the corresponding—new—procedural element. After the WTA net has made its decision and has activated the motivation unit of a procedure normally not used in the actual context, simulation using the internal body model will be started to test the contribution of this new procedure. Note that therefore a problem detector is also required inside the internal model which functions in the same way, i.e., it observes static stability of the (internally simulated) body (Figure 7, bold yellow arrows).

If during the internal simulation no problem detector becomes active, the procedure appears to be a suitable solution for the given problem. Thus, the solution is found following a search driven by a heuristic including noise (given to the SAL units). As a next step, this solution is tested for being mechanically appropriate. In this case the switch is set back to position 1 and the corresponding behavior will then be applied in reality. By setting back the switch the real body will provide the sensory input. As the winning WTA unit is still active (thus representing a short term memory), the newly selected procedure will be executed. If, however, already during the internal simulation this “new solution” has proven not to be a solution—defined by a problem detector of the internal model becoming active—the search for a solution will be continued further. To this end, the internal model will be reset to the current state of the body. Then, the SAL net will continue the spreading of its activations and a new behavior will be selected by the WTA-net. In this way the procedure will be repeated until a solution is found.

When the new solution is tested in reality, there are still two possibilities to be considered. If the realization of the proposed solution is successful, behavior continues. However, the solution may also turn out not to be realizable. This might for example happen because the body model does not simulate the physical properties of the body (and the environment) well enough. In this case a—possibly different—problem detector will be activated by starting again a new search procedure, with the internal body model being reset to the current real state of the body as given through the sensors.

In the remainder of this section, the structure that controls the temporal sequences sketched above is explained in detail. As indicated in the lower part of Figure 7, the complete procedure is controlled by five specific motivation units, Beh, SAL, WTA, SIM, and Test forming the center of the Global Phases network. These units are coupled via mutual inhibition (not depicted in Figure 7) and in part by transient, i.e., high-pass like, units (Figure 7, gray units and connections in the lower part).

During normal, i.e., reactively controlled walking the motivation unit “Beh” is active, thereby inhibiting the other four motivation units. If a problem is detected, the problem detector is activated which in turn inhibits the ongoing behavior (motivation unit “Beh”) and activates the “SAL” motivation unit. In addition, the switch is moved to bypass the physical body (the switch might be realized by further mutually coupled motivation units, not shown in Figure 7) and the current forward movement of the robot is inhibited for some time that corresponds to duration of about one step of the leg (i.e., 100 iterations). This allows sufficient time to test movements before starting to continue forward walking. After a given time required for sensible spreading of activations (e.g., two iterations, triggered by the “Delay” unit shown in gray in Figure 7), the SAL motivation unit is inhibited and the WTA motivation unit is activated instead. The relaxation of the WTA net may require a variable number of iterations. A simple solution is to introduce one unit observing the convergence of the WTA-network (“Relax”). This unit is activated as soon as the first unit of the WTA layer has reached a given threshold, representing the winning unit.

Only after a winner is detected (“Relax” in Figure 7), the “WTA” motivation unit is inhibited and the simulation is started (motivation unit “SIM”). If, after a given time of internal simulation (we use 400 iterations which equals 4 s or about three to four step cycles), no problem occurred, the motivation unit “Test” will be activated instead to start the real behavior. If during the test of the real behavior the problem occurs again or a new problem is detected (in contrast to the situation during simulation), the behavior is inhibited and the “SAL” motivation unit is activated again. If however the behavioral test is successful, too, the motivation unit “Beh” is activated (and the motivation unit “Test” inhibited) to allow continuation of the normal behavior. In contrast, if during simulation a problem is detected, the simulation is interrupted (motivation unit “SIM” is inhibited) and instead the motivation unit “SAL” is excited again to search for a new “idea.” The temporal order of activation of the different motivation units of the Global Phases network is controlled by dedicated connections running in parallel to the mutual inhibitory connections (Figure 7, gray) of all these units,

Importantly, each internal simulation has to start from the real situation, i.e., the situation that led to the problem. Therefore, the internal body model as well as the control system have to be reset to this state before a new internal simulation is started. This reset is triggered during the “SAL” stage. As the body did not actively move during internal simulation, the current posture and sensor readings can be used to reset the internal body model. It takes the reactive part of the control system only a couple (one or two) iterations to converge to the original state. It turned out that the internal state does not have to be stored explicitly.

The complete procedure controlled by the Global Phase network corresponds to what has been termed “incubation” and “verification” (Helie and Sun, 2010), and is similar to the “note-assess-guide” strategy or “metacognitive loop” as introduced by Anderson et al. (2006). In a mathematical analysis applied for example to logic reasoning systems the latter authors could show that introduction of such a strategy indeed improves the behavior of the complete system. The complete period, during which the body is fixed and the body model is used for internal simulation, may correspond to what Redish (2016), referring to Tolman, has termed “vicarious trial an error.”

## Results

### Simulation results for the example scenario

In this section, we will show a dynamic simulation of the reaCog system. The example illustrates the faculty of reaCog to find new solutions to a current problem using its capabilities for planning ahead. (In this study there is no physical robot used yet, but it is represented by a dynamic simulation.). Figure 6 shows an awkward posture. This configuration can become problematic as the left hind leg is already very far to the back and cannot move further back. Therefore, in this situation the left hind leg has to produce a swing movement. If the position of the left middle leg and right hind leg are positioned very far to the front, lifting the left hind leg can lead to instability, because the center of mass is placed quite far to the rear, between the hind legs. A sensible solution in our paradigm (Figure 6) might be the activation of the Swing_toBack module of the left middle leg: A backward step of the anterior middle leg might allow this leg to take over the body weight, and—as a consequence—afterwards allow lifting of the left hind leg. Thereby, continuation of walking may become possible.

In normal walking the reactive part of the controller is not ending up in such a strange posture. Therefore, we had to introduce an external disturbance to make the system tumble. To this end, the placements of the left middle leg and right hind leg will be changed in a way that during swing movement the target position is pushed further to the front (by a third of a step length). Such a strong change might occur in insects when climbing over irregular ground. When there is no foothold, the insects are starting searching movements to the anterior in order to find a foothold (Dürr and Krause, 2001; Bläsing and Cruse, 2004; Schütz and Dürr, 2011) which can be quite far to the front. This does not pose a problem for the stick insect as stability is strongly supported through the ability to attach the feet to the ground. As the robot cannot use this method, he has to find another solution (for example the one sketched in Figure 6).

**Figure 9:**
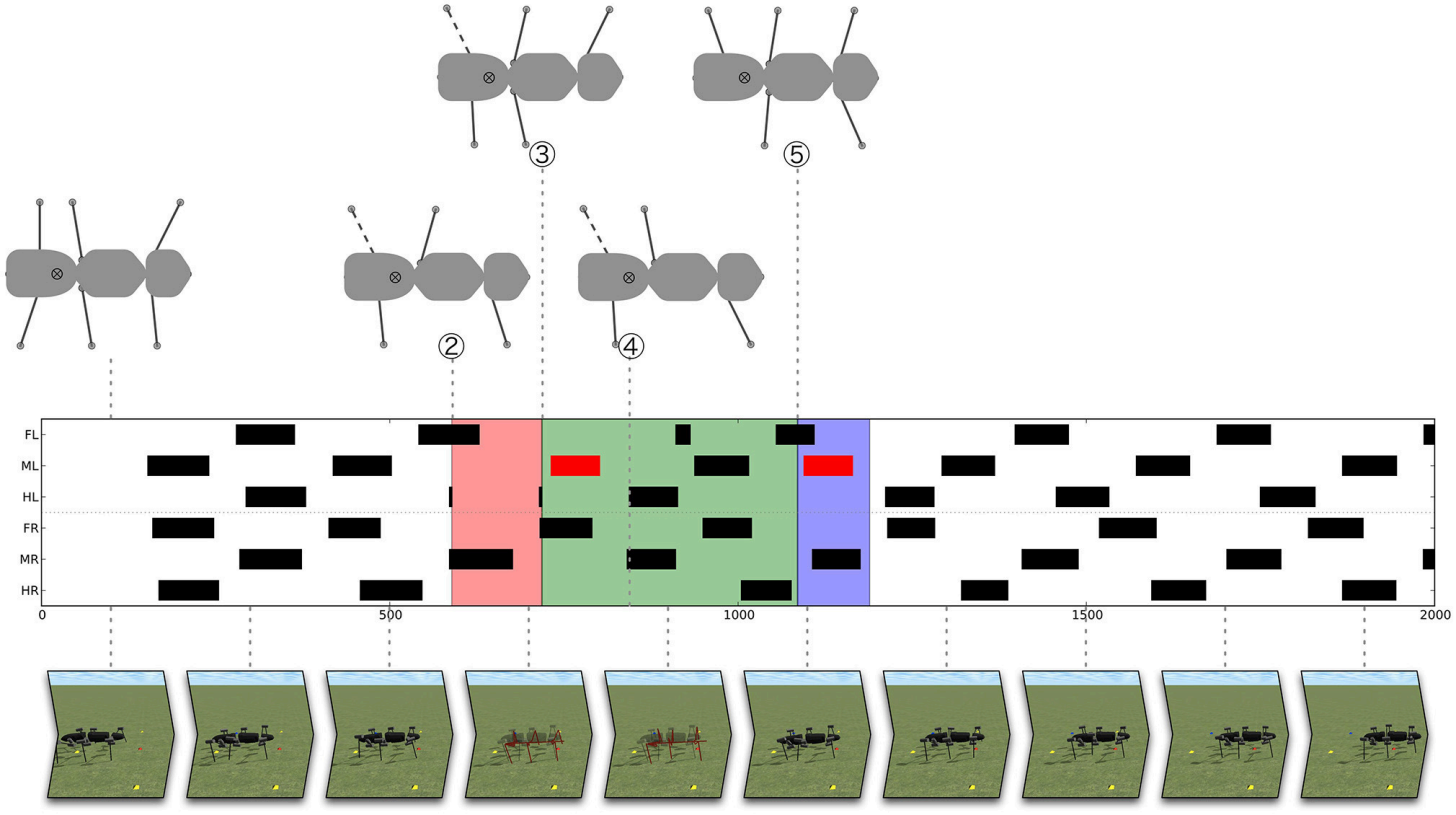
**Solving the problem illustrated in Figure 6: Foot fall patterns**. The middle panel shows the footfall pattern of the hexapod over time (black/red bars indicate swing movement of the leg). The upper panel shows some critical configurations of the robot (or, during internal simulation, the configuration of the internal model). The robot is walking from left to right. In three cases, the left hind leg is shown as a dashed line indicating that it is supposed to start a swing movement. The lower panel illustrates the behavior by screen shots taken from the Supplementary Material Videos 1, 2. The robot starts with a tripod-like leg configuration and converges to a fast tetrapod gait. The problem is detected at (#2). The section highlighted red represents an unsuccessful internal simulation [ending in an unstable configuration again as shown in (#3)]. The second internal simulation, highlighted green [starting at (#3)], turns out to be successful and solves the problem (backswing of the left middle leg, depicted by red bars, (#4) shows the new posture before the start of the forward swing movement of the left hind leg). Highlighted blue is the application of this solution to the robot (starting at (#5) which shows the robot posture at the beginning of the backward swing movement of the left middle leg). This final test is successful, too, and the robot continues to walk (⊗ indicates center of mass).

**Figure 10:**
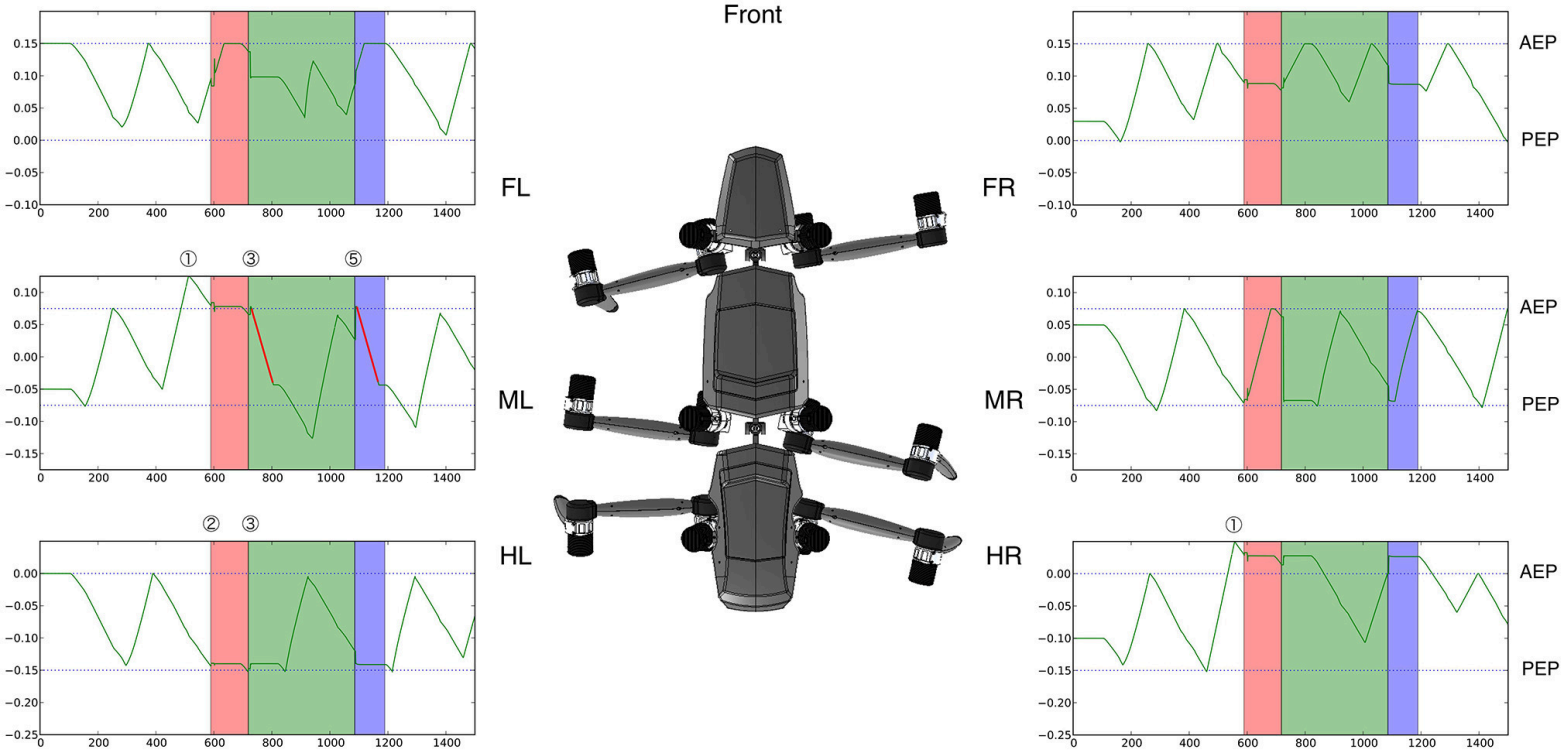
**Solving the problem: Position of the individual legs over time**. Green lines show the position of each leg over time—positive values are toward the front of the walker. Ordinate is given in cm with the origin fixed to the COM of the robot. The blue dashed lines indicate the average extreme positions: The Anterior Extreme Position (AEP) is the target position for the swing movement and is fixed during forward walking. The Posterior Extreme Position (PEP) indicates the position at which a leg controller initiates a swing movement on average and switches from stance to swing (note that the coordination rules act on the PEP and shift the PEP forward or rearward to organize the overall behavior which is not shown in the figure). Shortly after the left middle and right hind leg performed swing movements that point very far to the front of the working range (#1), the walker becomes unstable (#2) when trying to lift the left hind leg. Therefore, internal simulations are started (highlighted in green and red) during which motor commands are routed to the internal body model, the leg positions of which are shown. First (highlighted red), an unsuccessful behavior is tested: a stance movement which has initially no effect as the agent is stopped. But when the agent accelerates again (after 100 iterations) the problem is still present and the agent becomes instable (#3). As a second trial, a backward swing movement of the middle left leg is tested via internal simulation (green highlighted area; the swing movement in the unusual direction is plotted in red). Afterwards (#5) the solution found is tested on the real robot (highlighted in blue) showing that walking continues successfully.

In the following, with help from Figures 9, 10, we will explain how the system deals with this intervention. Figure 9 (middle panel) shows a footfall pattern which illustrates the swing movements of the legs over time. A leg which is in swing phase is marked as a black (or red) bar. For the medium velocity chosen a gait is emerging that can be seen in the stepping pattern in the left part of the figure. From a tripod-like starting posture the robot converges more toward a fast tetrapod-like gait (at about 500 iterations). The lower part of Figure 9 shows still images of the dynamic simulation (see Supplementary Material Videos 1, 2), whereas the upper part provides a top view of the robots' (or internal models') configuration. The upper part shows four specific snapshots of the posture of the walker (top view) facing to the right. Only legs in stance phase, i.e., legs which support stability are depicted.

For the same run, Figure 10 illustrates the position of each leg over time. The position is plotted on the ordinate showing the movement of the leg (green lines, swing movements during forward walking are pointing upwards; stance movements are going into the opposite direction).

The jumps in the position of the legs are due to the switching from the real robot to the internal model required to reset the internal model. Colors are used as in Figure 9. For further explanations see text.

As mentioned, we forced the robot into an awkward posture in such a way that the swing movement of the left middle and right hind leg moved very far to the front of their working range, i.e., beyond their normal AEP. Next, the left hind leg marked by a dashed line in Figure 9 is supposed to start a swing movement. The center of mass would then not be supported anymore by the left middle leg and right hind leg [Figures 9, 10 (2), after 580 iterations]. Therefore, the system would tumble backwards.

As a consequence, the problem detector is activated, which stops the overall movement of the robot and triggers the cognitive expansion which then starts motor planning. In the example shown in Figures 9, 10 the robot first selects a stance movement in the left hind leg (due to the somatotopical neighborhood, see Figure 7, in SAL layer). This stance movement is then applied in internal simulation.

As a result, an unsuccessful internal simulation can be observed (highlighted in red) (2)–(3), which is interrupted when the left hind leg should be lifted, because this action would again lead to an instable configuration of the internal body model [see upper panel, (3)]. Note that during the time highlighted in red (and green, see below) the robot is not moving. Only the internal model is used to provide predictions of the movements.

As a consequence, a second iteration of the cognitive expansion is invoked (this section is highlighted green, as it turns out to be successful): First, activation is further spread in the SAL layer. Second, a behavior is selected in the WTA layer which has not yet been tested. And third, the behavior is applied as internal simulation.

For this second internal simulation, the internal body model and control system have to be reset initially. To this end, it turned out to be sufficient to update, first, the internal model with the values from the real robot structures (this is the starting condition required for the internal simulations; see Figure 10, at the border of the red and green section, the position of the leg in the internal model jumps back to the original position of the robot leg). Second, as the control system is behavior-based it depends on the sensor state represented by the current position of the robot. This state can be enforced onto the control system so that the system converges back to its behavioral state.

In the simulation run shown, the behavior selected next is a backward swing movement of the left middle leg (depicted in Figure 10 by a red line for the position of the left middle leg; correspondingly, in Figure 9 the swing movement backwards is shown as a red bar). As illustrated in the parts highlighted in green, again the forward movement of the body is interrupted for some time. During this time the newly selected behavior is tested by internal simulation. When the system starts to accelerate again, the left middle leg now being placed further to the rear helps to support the robot. When the left hind leg starts to swing, the left middle leg is ready to take over and to support the body (shown in Figure 9 in the upper panel in the body posture at #4 at around 800 iterations). The internal simulation runs further for a given time (here we used additional 300 iterations) in order to guarantee that normal walking can be continued.

When the internal simulation was successful the behavior selected (which is still stored in the WTA layer) will be applied on the (simulated) physical system (see #5 and blue area in Figures 9, 10). This part is still regarded as a test of the behavior. This test is necessary because internal simulation and robot can of course lead to slightly different results which over time might accumulate. For example, in Figure 9 the behavior of the right middle leg differs between internal simulation and testing the behavior on the robot. The right middle leg is very close to its posterior extreme position and on the verge of starting a swing movement. In both cases, the robot is standing still and not supposed to move further forward. But in the case of the robot (highlighted blue), a small passive movement would be sufficient to initiate a swing movement. Nonetheless, as can be seen from the footfall pattern, the application on the robot is also successful and the system converges to a stable gait pattern. This stresses the robustness of the underlying control approach and highlights how important it is that planning and control are tightly intertwined. In the blue area and beyond, Figure 10 shows the movements of the leg of the real robot. Immediately after the new behavior has been induced, one can observe how the phases of the individual leg controllers are rearranged. For example, the right front leg is forced to make an early swing movement after the right middle leg has finished its swing movement (see Schilling et al., 2013b). But already after a very short time, a single step of the robot, a stable tetrapod-like gait emerges (as can be seen in Figure 9).

The example illustrates the faculty of reaCog to activate behavioral elements out of context in order to find a solution to a current problem. As illustrated, the system (reaCog plus robot) manifests an impressive stable behavior, although various deviations from normal walking behavior can be observed during the complete process.

### Simulation series on disturbed walking

For a more quantitative evaluation of the performance of the reaCog architecture we performed two additional series of simulations to illustrate the contributions of the different parts of the system. On the one hand, there is the underlying reactive and biological inspired control system (based on Walknet Schilling et al., 2013a). On the other hand, when running into stability problems the cognitive expansion has been introduced which can take over in order to reconfigure the posture in a way that allows to continue stable walking.

Following the approach presented above in detail, we again used the repositioning of a leg during swing movement which means that, for a selected swing movement, the target position is shifted to the front. This represents a quite natural example disturbance as the insects are often climbing through twigs that do not provide many footholds. As a consequence, insects perform searching movements that may shift the end position of the swing movements further to the front.

As a first series of simulations, after a randomly chosen point in time (during the first 10 s of walking) one leg is selected randomly for which the next swing movement is shifted to the front (about 5 cm which equals a third of a complete step length). In this way, different legs are affected in different walking situations. We ran 100 different simulations, therefore each leg was targeted multiple times and in the different stages of the 10 s of walking. As a result, when only one leg is targeted the reactive control system showed to be sufficient and the walker never got unstable independent of which leg was shifted. For all simulations, walking continued for at least 5 more seconds after the disturbance. In most cases, already after one subsequent step the control system has established again a stable walking pattern. Only for an early change in a front leg this requires two stepping cycles. Stability is accomplished mainly through compensating the leg shift. While the shifting of the target position would prolong the next step for the respective leg, the local coordination influences force the leg into an earlier lift-off in order to compensate. Detailed results are provided as Supplemental Data 1 in Supplementary Material which show for each of the different legs (front, middle, and hind leg) a single run as an example. As can also be seen in the data, the walking pattern emerges quite early in the first or the second step.

As a more severe disturbance, we performed a series of simulations in which two legs were targeted. Again, after a randomly chosen point in time (during the first 10 s of walking) two legs are selected randomly for which the next swing movement is shifted to the front (about 5 cm which equals a third of a complete step length). We performed 100 simulation runs with all kind of combinations between legs multiple times. As already discussed for the example shown above (Section Simulation Results for the Example Scenario), in this case the reactive biologically inspired control system may run into unstable situations that require to stop the walking behavior to avoid that the robot would topple over. In the following we provide results on for how many cases the system struggled with stability and how the cognitive expansion was able to deal with those situations. Overall, there are eight instable situations which were caused by a disturbance of a middle and the diagonal hind leg (a case as described in detail above, Section Simulation Results for the Example Scenario). For these eight simulation runs the cognitive expansion had to take over and has found a solution in all instances. The system always became instable when the other (non-disturbed) hind leg tried to initiate a swing movement. Interestingly, different solutions have been found. On the one hand, a rearrangement of the legs could be observed in a way that one leg was moved backwards and unload the non-disturbed hind leg which afterwards was able to initiate a swing movement. This was accomplished either through moving backwards the anterior middle leg or the contra lateral hind leg. On the other hand, we observed two cases in which the slowing down of the walking speed of the complete system was already sufficient to solve the problem as during the slowing down a swing movement could be terminated which provided enough support for the walker.

These results show that the cognitive expansion is able to find different suitable solutions. Note, that the solution disrupts the coordination pattern of all the legs. Only together with the reactive system and the coordination rules the system is able to select a movement which enables stable ongoing walking. In some instances the system discarded solutions which we, on a first guess, would have assessed as possible solutions, but which later-on run into conflicts.

## Related work

In this section, we will compare reaCog as a system with related recent approaches in order to point out differences. While there are many approaches toward cognitive systems and many proposals concerning cognitive architectures, we will concentrate on models that, like reaCog, consider a whole systems approach. First, we will deal with cognitive architectures in general. Second, we will briefly present relevant literature concerning comparable approaches in robotics, because a crucial property of reaCog is that it uses an embodied control structure to run a robot.

### Models of cognitive systems

Models of cognitive systems generally address selected aspects of cognition and often focus on specific findings from cognitive experiments (e.g., with respect to memory, attention, spatial imagery; review see Langley et al. (2009), Wintermute (2012). Duch et al. (2008) introduced a distinction between different cognitive architectures. First, these authors identified symbolic approaches. As an example, the original SOAR (State, Operator, and Result; Laird, 2008) has to be noted, a rule-based system in which knowledge is encoded in production rules that allow to state information or derive new knowledge through application of the rules. Second, emergent approaches follow a general bottom-up approach and often start from a connectionist representation. As one example, following a bottom-up approach, Verschure et al. (2003) introduced the DAC (Distributed Adaptive Control) series of robot architectures (Verschure et al., 2003; Verschure and Althaus, 2003). These authors initiated a sequence of experiments in simulation and in real implementation. Verschure started from a reflex-like system and introduced higher levels of control on top of the existing ones which modulated the lower levels and which were subsequently in charge on longer timespans (also introducing memory into the system) and were integrating additional sensory information. The experiments showed that the robots became more adapted to their environment exploiting visual cues for orienting and navigation etc., (Verschure et al., 2003). Many other approaches in emergent systems concentrate on perception, for example, the Neurally Organized Mobile Adaptive Device (NOMAD) which is based on Edelman (1993) Neural Darwinism approach and demonstrates pattern recognition in a mobile robot platform (Krichmar and Snook, 2002). Recently, this has gained broader support in the area of autonomous mental development (Weng et al., 2001) and has established the field of developmental robotics (Cangelosi and Schlesinger, 2015). A particular focus in such architectures concerning learning is currently not covered in reaCog. In general, as pointed out by Langley et al. (2009), these kinds of approaches have not yet demonstrated the broad functionality associated with cognitive architectures (and—as in addition mentioned by Duch et al. (2008)—many of such models are not realized and are often not detailed enough to be implemented as a cognitive system). ReaCog realizes such an emergent system but with focus on a complex behaving system that, in particular, aims at higher cognitive abilities currently not reached by such emergent systems. The third type concerns hybrid approaches which try to bring together the advantages of the other two paradigms, for example ACT-R (Adaptive Components of Thought-Rational, Anderson, 2003). The, in our view, most impressive and comprehensive model of such a cognitive system is presented by the CLARION system (review see Sun et al., 2005; Helie and Sun, 2010) being applied to creative problem solving. This system is detailed enough so that it can be implemented computationally. Applying the so called Explicit-Implicit Interaction (EII) theory and being implemented in the CLARION framework, this system can deal with a number of quantitatively and qualitatively known human data, by far more than can be simulated by our approach as reaCog, in contrast, does not deal with symbolic/verbal information. Apart from this aspect, the basic difference is that the EII/CLARION system comprises a hybrid system as it consists of two modules, the explicit knowledge module and the implicit knowledge module. Whereas, the latter contains knowledge that is not “consciously accessible” in principle, the explicit network contains knowledge that may be accessible. Information may be redundantly stored in both subsystems. Mutual coupling between both modules allows for mutual support when looking for a solution to a problem. In our approach, instead of using representational differences for implicit and explicit knowledge to cope with the different accessibility, we use only one type of representation, that, however, can be differently activated, either being in the reactive mode or in the “attended” mode. In our case, the localist information (motivational units) and the distributed information (procedural networks) are not separated into two modules, but form a common, decentralized structure. In this way, the reaCog system realizes the idea of recruitment as the same clusters are used in motor tasks and cognitive tasks. Whereas, we need an explicit attention system, as given in the spreading activation and winner-take-all layer, in the CLARION model decisions result from the recurrent network finding an attractor state.

Many models of cognition take, quite in contrast to our approach, as a starting point the anatomy of the human brain. A prominent example is the GNOSIS project (Taylor and Zwaan, 2009). It deals with comparatively fine-grained assumptions on functional properties of brain modules, relying on imaging studies as well as on specific neurophysiological data. While GNOSIS concentrates mainly on perceptual, in particular visual input, the motor aspect is somewhat underrepresented. GNOSIS shows the ability to find new solutions to a problem, including the introduction of intermediate goals. Although an attention system is applied, this is used for controlling perception, not for supporting the search, as is the case in reaCog. Related to this, the search procedure—termed non-linguistic reasoning—in GNOSIS appears to be less open as the corresponding network is tailored to the actual problem to avoid a too large search space. In our approach, using the attention system, the complete memory can be used as substrate for finding a solution.4.2 Cognitive Robotic Approaches

The approaches introduced in the previous section are not embodied and it appears difficult to envision how they could be embodied (Duch et al., 2008). Following the basic idea of embodied cognition (Brooks, 1989; Barsalou, 2008; Barsalou et al., 2012) embodiment is assumed as being necessary for any cognitive system. Our approach toward a minimal cognitive system is based on this core assumption. Robotic approaches have been proposed as ideal tools for research on cognition as the focus cannot narrowed down to a singular cognitive phenomenon, but it is required to put a unified system into the full context of different control processes and in interaction with the environment (Pezzulo et al., 2012).

ReaCog as a system is clearly embodied. The procedures cannot by themselves instantiate the behavior, but require a body. The body is a constitutive part of the computational system, because the sensory feedback from the body is crucially required to activate the procedural memories in the appropriate way. The overall behavior emerges from the interaction between controller, body and environment. In the following, we will review relevant embodied robotic approaches.

Today, many robotic approaches deal with the task of learning behaviors. In particular, behaviors should be adaptive. This means, a learned behavior should be transferable to similar movements and applicable in a broader context. Deep learning approaches have proven quite successful in such tasks e.g., Lenz et al. (2015), but many require large datasets for learning. Only recently Levine et al. (2015) presented a powerful reinforcement learning approach in this area. In this approach, the robot uses trial-and-error during online learning to explore possible behaviors. This allows the robot to quickly learn control policies for manipulation skills and has shown to be effective for quite difficult manipulation tasks. When using deep learning methods it is generally difficult to access the learned model. In contrast to reaCog such internal models are therefore not well suited for recruitment in higher-level tasks and planning ahead. In particular, there is no explicit internal body model which could be recruited. Rather, only implicit models are learned and have to be completely acquired anew for every single behavior.

In the following, two exciting robotic examples tightly related to our approach will be addressed in more detail. The approach by Cully et al. (2015) aims at solving similar tasks as reaCog for a hexapod robot. It also applies as a general mechanism the idea of trial-and-error learning when the robot encounters a novel situation. In their case these new situations are walking up a slope or losing a leg. There are some differences compared to reaCog. Most notably, the testing of novel behaviors is done on the real robot. This is possible as the trial-and-error method is not applying discrete behaviors. Instead, central to the approach by Cully et al. (2015) is the idea of a behavioral parametrization which allows to characterize the currently experienced situation in a continuous, low dimensional space. A complete mapping toward optimal behaviors has been constructed in advance offline (Mouret and Clune, 2015). This pre-computed behaviors are exploited when a new situation or problem is encountered. As the behavioral space is continuous, the pre-computed behavior can be used to adapt for finding a new behavior. Further, there is no explicit body model that is shared between different behaviors. Instead, the memory approximates an incomplete body model, as it contains only a limited range of those movements which are geometrically possible. In contrast, reaCog, using its internal body model, allows to exploit all geometrically possible solutions and is not constraint to search in a continuous space, as illustrated by our example case, where a single leg is selected to perform completely out of context.

While there is only a small number of robotic approaches dealing with explicit internal simulation, most of these are using very simple robotic architectures with only a very small number of degrees of freedom [for example see Svensson et al. (2009) or Chersi et al. (2013)]. It should further be mentioned that predictive models are also used to anticipate the visual effects of the robot's movements (e.g., Hoffmann, 2007; Möller and Schenck, 2008). With respect to reaCog the most similar approach has been pursued by Bongard et al. (2006). These authors use a four-legged, eight DoFs robot which, through motor babbling—i.e., randomly selected motor commands—learns the relation between motor output and the sensory consequences. This information is used to distinguish between a limited number of given hypotheses concerning the possible structure of the body. Finding the best fitting solution, one body model is selected. After the body model has been learned, in a second step the robot learns to move. To this end, the body model was used to perform different simulated behaviors and was only used as a forward model. Based on a reward given by an external supervisor and an optimizing algorithm, the best controller (sequence of moving the eight joints) was then realized to run the robot. Continuous learning allows the robot to register changes in the body morphology and to update its body model correspondingly. As the most important difference, Bongard et al. (2006) distinguish between the reactive system and the internal predictive body model. The central idea of their approach is that both are learned in distinct phases one after another. In reaCog the body model is part of the reactive system and required for the control of behavior. This allows different controllers driving the same body part and using the same body model for different functions (e.g., using a limb as a leg or as a gripper, Schilling et al., 2013a, Figure 10). In addition, different from our approach, Bongard et al. (2006) do not use artificial neural networks (ANN) for the body model and for the controller, but an explicit representation because application of ANN would make it “difficult to assess the correctness of the model” (Bongard et al., 2006, p. 1119). ReaCog deals with a much more complex structure as it deals with 18 DoFs instead of the only eight DoFs used by Bongard et al. (2006) which makes an explicit representation even more problematic.

Different from their approach, we do not consider how the body model and the basic controllers will be learned, but take both as given (or “innate”). While the notion of innate body representations is controversial (de Vignemont, 2010), there is at least a general consensus about that there is some form of innate body model (often referred to as the body schema) reflecting general structural and dynamic properties of the body (Carruthers, 2008) which is shaped and develops further during maturation. This aspect is captured by our body model that encodes general structural relations of the body in service for motor control, but may adapt to developmental changes. While currently only kinematic properties are applied, dynamic influences can be integrated in the model as has been shown in Schilling (2009).

A further important difference concerns the structure of the memory. Whereas, in Bongard's approach one monolithic controller is learned to deal with eight DoFs and producing one specific behavior, in reaCog the controller consists of modularized procedural memories. This memory architecture allows for selection between different states and therefore between different behaviors.

## Discussion and conclusion

A network termed reaCog has been proposed that is based on the reactive controller Walknet equipped with decentrally organized behavioral modules, or procedures, all connected to motivation units, and a body model. The motivation units form a network that represents a heterarchical architecture allowing for the realization of various internal states. These states result from parallel activation of elements as well as competitive selection between elements.

The body model can be used as an inverse model for controlling motor output, as a forward model for internal simulation of behavior, and it can be exploited to improve erroneous sensor data (“sensor fusion”). Whereas, the reactive part uses the ability of the body model to function as an inverse model, the cognitive expansion exploits the internal body model to be used as a forward model and thereby as a tool for internal simulation of behavior. Internal simulation is used for finding a new solution for a problem detected by a problem detector. To this end, a three-layered network has been introduced that selects a new, currently not used module of the procedural memory, the contribution of which will then be tested through internal simulation. If this simulation turns out to be successful, i.e., shows a solution for the current problem, the corresponding behavior will be executed in reality. Thus, motor planning is possible using an extremely small expansion, a network consisting of essentially six units plus three parallel layers of units connected in a simple way.

In reaCog, there is no explicit, separate planner as used in hybrid systems. Rather, the ability to plan ahead relies on exploiting the reactive basis by operating on it much like a parasite operates on its host, that is, by only controlling the functioning of the reactive part. In other words, the cognitive expansion does not represent a separate planner, but organizes the activity of the reactive part, which is, during planning, not connected with the motor output.

Thus, constitutive elements of reaCog are (1) embodiment, (2) a decentralized organization of various procedures arranged in a heterarchical architecture, (3) a holistic body model allowing for pattern completion that is used in reactive behavior and can be recruited for planning ahead, and (4) a small network, called cognitive expansion, that enables the otherwise reactive system to become—in the sense of McFarland and Bösser (1993)—a cognitive one. We are not aware of any other neuronal approach that covers all these properties. Although the network represents a simple architecture, in the following we will argue that properties often attributed to “higher” brains can be found in reaCog, too, thereby approaching the question concerning the basic neuronal requirements of such higher level phenomena.

Before entering into this discussion, one important aspect missing in the current version of reaCog has to be noted. There is long term memory represented by the procedures in the form of “species memory” (Fuster, 1995). There is short term memory as a new solution is stored until the corresponding behavior is executed. There is however no faculty yet to transform the content of this short term memory into a long term memory. The ability to store such a newly acquired procedure as a long term memory would of course be an advantageous property. To gain this capability, the sensory situation accompanying the occurrence of a “problem” should be able to directly elicit activation of the procedure found to solve the problem.

When discussing the properties of a network like reaCog, a crucial aspect concerns the notion of emergence. The rational behind searching for emergent properties is the assumption that many “higher level” properties are not based on dedicated neuronal systems specifically responsible for the respective properties. Rather, emergent properties arise from the cooperation of lower-level elements and are characterized as to require levels of description other than those used to describe the properties of the elements. In the remainder, such emergent properties will, where appropriate, be related to the requirements posed by Langley et al. (2009) supporting the idea that reaCog provides a minimal functional description for some of those requirements.

According to Langley et al. (2009), a cognitive system should show the following properties: (1) Storing motor skills and covering the continuum from fully reactive, closed-loop behavior to (automatic) open-loop behavior; (2) Emergent properties resulting from the cooperation between different independent modules; (3) Long term memory and short term memory; (4) Attention to select sensory input; (5) Decisions on the lower level and “choice” on the higher level; (6) Predictions of possible actions; (7) Problem solving and planning of actions in the world; (8) Recognition and categorization of sensory input; (9) Remembering and episodic memory; (10) Application of symbols and reasoning; (11) To support reasoning, relationships between beliefs have to be realized; (12) Interaction and communication, including representation of verbal symbols; (13) Reflection and explanation (metareasoning); (14) Confronting the interactions between body and mind.

Requirements (1) and (2) are properties of Walknet. Above, we already argued that requirements (3), (6), and (7) can be found in reaCog, too. Below we will argue that also requirements (4) and (5) are fulfilled, but not aspects (8)–(14).

As the cognitive expansion of the reactive network allows the complete system—using psychological terms to describe its function—to “focus” or “concentrate” or “attend” on a specific behavior, we have already earlier termed this expansion “attention system” supporting Langley et al.'s issue (4). Its ability to focus on specific memory elements may correspond to what sometimes has been termed “spot light” (Baars and Franklin, 2007) referring to the observation that the content of only one memory element becomes aware at a given moment in time. Recall, that selection of a specific procedure via the WTA network of the attention system does not mean that the other procedures are suppressed. The cognitive expansion network does not prohibit parallel activation of procedures. This requirement is in line with current developments in the area of cognitive systems research as pointed out by Duch et al. (2008). Inspired by the way how brains are organized, these authors propose, first, that cognitive systems in the future should incorporate a mechanism to focus attention, which is realized in reaCog through simple local competition as realized in the WTA structures. And second, that a neural network-like spreading activation mechanism is required in order to broaden search and follow associations, which is given in the spreading activation layer.

The fifth aspect of Langley et al. (2009) is concerned with action selection on lower levels and “choice” of behavior on a higher level. Action selection is indeed a crucial property of the network. On a lower level, within a given behavioral context—in our case walking—specific procedures compete via local WTA connections. For instance, a leg controller has to decide when to perform swing or stance movements. On an intermediate level, a decision can be made between, for example, forward walking and backward walking. On an even higher level, reaCog, exploiting the cognitive expansion, can select one specific behavioral element to be activated in addition to the currently active units. Therefore, Langley et al.'s requirement (5) is covered, too.

Thus, reaCog shows action selection not only on the reactive level, but also on the cognitive level, whereby the decisions based on internal simulation (or imagined action, “mental” action, or “probehandeln” according to Freud (1911) are not determined strictly by the sensorily given situation. Even if an external observer had the ability to monitor the internal states of the agent controlled by reaCog, the behavior of the agent could not be predicted by this observer. This is the case because, due to the noise in the SAL network, there is a stochastic element contributing to the decision. On the other hand, the final decision is not purely random, because the proposals made by the attention system are tested for feasibility via the internal simulation and are to some extent guided by the somatotopic structure of the SAL network. The proposal is further tested by performing the behavior in reality. In this way, this process of finding a new solution may be viewed as to be based on a Darwinian procedure, starting with an, in part, stochastic “mutation,” followed by a selection testing the proposal for “fitness.”

It has been stated that in a cognitive system, in order to address memory elements out of context (“global availability,” e.g., Dehaene and Changeux, 2011), these elements have to be represented independently, i.e., not embedded in reactive structures. In reaCog, procedures are not represented independently, but are always represented within their context. Nonetheless, the functioning of the cognitive expansion allows to integrate them in another context. In other words, in reaCog, the procedures are globally available. Therefore, global availability may not require procedures being stored independent of any context (or “amodally”). Thus, reaCog represents a concrete architecture showing how global availability might be established in a neural system without requiring independent representation.

There is a group of related terms addressing a fundamental principle of brains. These are the “massive redeployment hypothesis” (Anderson, 2010), the “neural recycling theory” (Dehaene, 2005), the “shared circuits model” of Hurley (2008) and Gallese's “neural exploitation” (Gallese and Lakoff, 2005), summarized by Anderson (2010) by the term “neural reuse.” Neural reuse means that a system is able to exploit existing components that do something useful to support a new task, either in the evolutionary time frame or by learning (Anderson, 2010, p. 250). In other words, neural reuse states that existing elements are used for other purposes. ReaCog models neural reuse of two kinds as listed by Anderson. One type, already applied in the current version of reaCog, corresponds to the use of the same procedural elements for both motor control and planning. Here reuse corresponds to the case of having been installed in evolutionary time scales. The second type addressable in reaCog concerns the reuse of procedural elements as a result of learning the integration of a given procedure in a new context as described above, which is, in reaCog, currently only given in the form of short term memory. But the ability to transfer this information into long term memory is a major focus for future work.

Although the structure of reaCog is far away from any morphological similarity to mammalian brains, functionally reaCog shows some similarity and may, therefore, in spite of its simplicity, be considered as a scaffold helpful for the understanding of properties of higher brains. To this end, taking a more abstract view, one might ask whether higher level properties characterized by using psychological terminology might be attributed to reaCog. As noted earlier, in reaCog emergent properties can already be observed at lower levels (e.g., production of different gaits) but they can also be found at higher levels, thereby supporting Langley et al.'s second requirement. Above, we had already used one such higher level term, attention. It has been argued (Cruse and Schilling, 2013, 2015) that further emergent properties as are intentions and emotions might be attributed to a system as reaCog, too, at least on the functional level. When adding some further procedures, reaCog might even be equipped with basic aspects required for Access Consciousness as well as Reflexive Consciousness (Cruse and Schilling, 2013, 2015).

Taken together, Langley et al.'s (Langley et al., 2009) requirements for cognitive systems (1)–(7) are well covered by reaCog. To conclude, we will briefly address the remaining issues (some of which have already been mentioned above): The capability to categorize sensory input, [Langley et al.'s issue (8)] is not given in reaCog as we focus mainly on the motor aspects. As mentioned, learning will be the focus of future work and will address episodic memory (9). Other aspects would require further extension: Langley et al.'s issues (10)–(12) refer to the ability to use (verbal) symbols, a property not given in reaCog. However, a way has been sketched how this might be possible (for a first step toward this property see (Schilling and Spranger, 2010; Cruse and Schilling, 2013; Schilling and Narayanan, 2013), based on ideas of Steels and Belpaeme (2005) and Narayanan (1999).

Langley et al.'s issue (13) concerns Reflection and explanation. This property is not realized in reaCog and may also depend on the ability to apply symbolic knowledge. Issue (14), the property of cognitive systems to “confront the interactions between body and mind” addresses the property of having phenomenal experience, and is not found in reaCog, too (for a discussion of this matter see Cruse and Schilling, 2013, 2015). In summary, a number of emergent properties can be observed in reaCog, including Langley et al.'s issues (1)–(7). Issues (8)–(13) require further expansions.

In this article we focus on the situation that there is a problem which requires immediate solution and as a consequence, immediate internal simulation. As in our situation the body model is needed for simulation, the former cannot be used for controlling other behaviors at the same time. In other words, the body position has to be kept constant during internal simulation. In the following we briefly mention three cases which do not comply with this situation. In the first case internal simulation is not required. In this simple case the network is equipped with reactive procedures that allow for unspecific, general responses in case a problem detector is activated. An ubiquitous example is given by freezing behavior without active search for a solution, hoping that the problem will disappear on its own. Another example might be a procedure that allows emitting a general alarm signal that activates conspecifics. As a second case, one might think of situations that allow to postpone the search for a solution. In this case the normal behavior can be continued until a situation is given that allows to use the internal model without getting into conflict with current behavior. This case would at least require a short term memory to store the problem situation so that this could later be reactivated to start internal simulation, an expansion not yet implemented in the current version of reaCog. As a third, more complex case there might be a network that is able to control any behavior and at the same time, run an internal simulation. Whereas, for the second case a comparatively simple expansion of reaCog would suffice, the latter case appears to be much more demanding. It might, for example, require a second internal model plus the corresponding circuit to control both models independently.

The term “cognition” as used here, is based on the simple definition proposed by McFarland and Bösser (1993), i.e., the faculty of being able to plan ahead. This faculty is achieved here by using a reactive system plus introduction of a “cognitive expansion.” As discussed above, such a system appears to be suited to form a basis on which further emergent properties may be realized, properties that are often listed as being required for a system termed cognitive as are Langley et al's requirements (8)–(14), for example. If this view is correct, these properties need not necessarily be explicitly included in such a definition, but appear to result from a system based on reactive structures plus the critical capability of planning ahead, underlining the power of McFarland and Bösser (1993) clear-cut definition of cognition.

## Author contributions

Conceptualization, methodology and writing—MS and HC. Software, investigation and simulation—MS.

### Conflict of interest statement

The authors declare that the research was conducted in the absence of any commercial or financial relationships that could be construed as a potential conflict of interest.

## Supplementary material

Supplemental Data 1 **Showing example runs from additional simulations**.

Supplementary Video 1 **AwkwardPosture_InternalSimulation.mp4**. Video showing the example case of testing different behaviors out of context in internal simulation and afterwards applying a successful solution to the robot.

Supplementary Video 2 **AwkwardPosture_InternalSimulation_LegPositions.mp4**. Video showing the example case of testing different behaviors out of context in internal simulation and afterwards applying a successful solution to the robot. Showing the leg positions over time.
