# Supplementary material for: ReaCog, a Minimal Cognitive Controller Based on Recruitment of Reactive Systems
Source: Front Neurorobot. 2017 Jan 30;11:3. doi: 10.3389/fnbot.2017.00003 (PMC5276858; doi:10.3389/fnbot.2017.00003)

Supplemental Material 1:
Single leg random AEP shift

For the following simulations a randomly chosen swing movement for a single leg is shifted in anterior direction. Shown are three examples for front, middle and hind leg.

A – Early front leg disturbance

Footfall pattern for early front left leg disturbance (first swing movement):

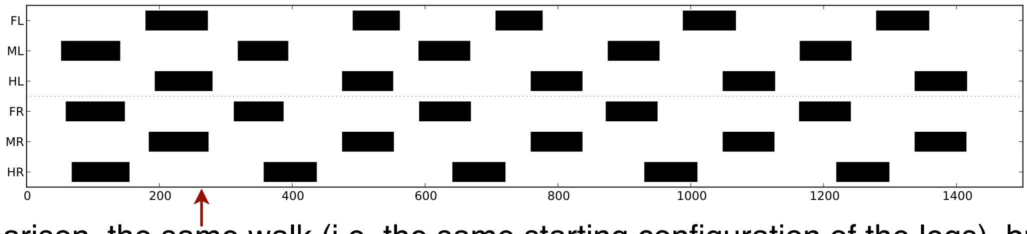

For comparison, the same walk (i.e. the same starting configuration of the legs), but without the disturbance.

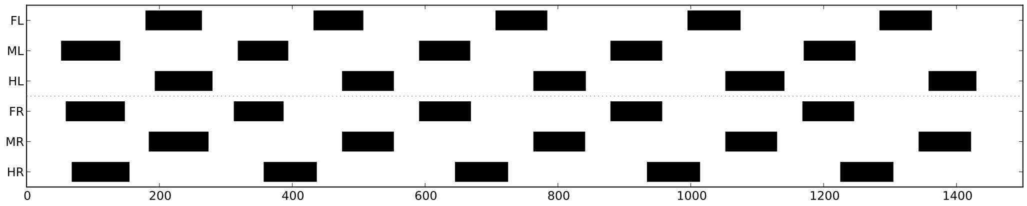

Leg position over time (shown is leg movement from posterior (negative y values) to anterior (positive y values) over time. The arrow marks the shift of the front left leg (upper left) to the front for the first swing movement. This is compensated during the next two steps with shortened stance movements:

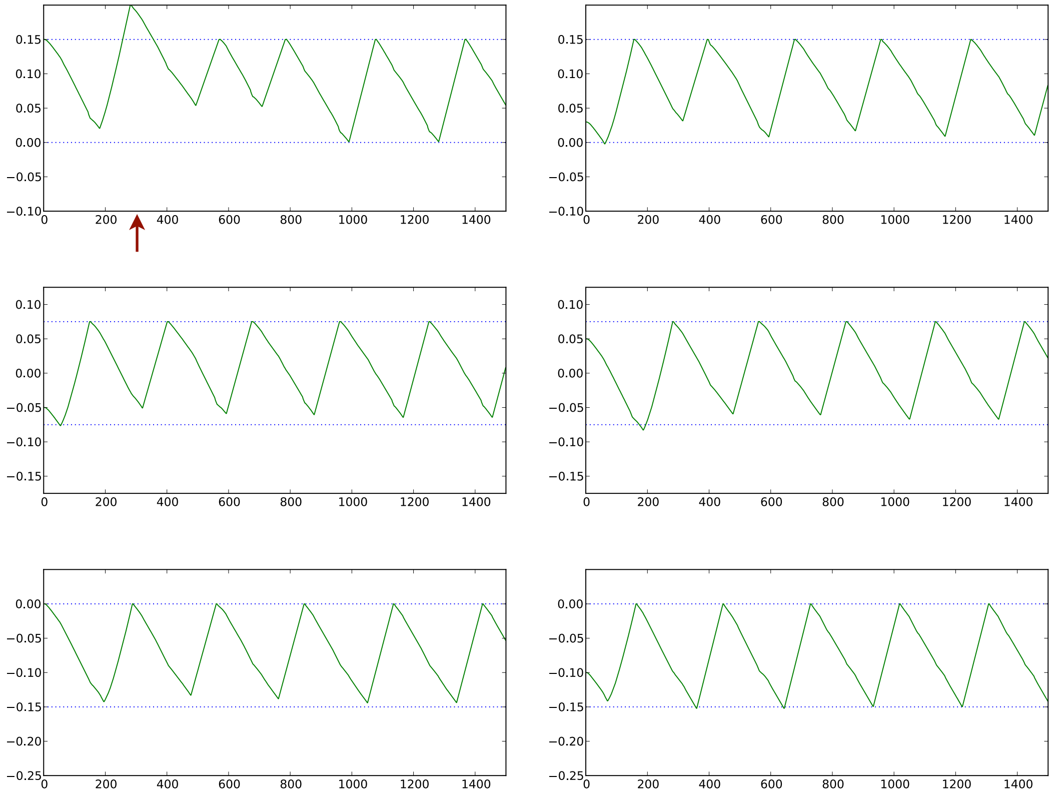

B – Late middle leg disturbance

Footfall pattern for late middle left leg disturbance (after nine seconds):

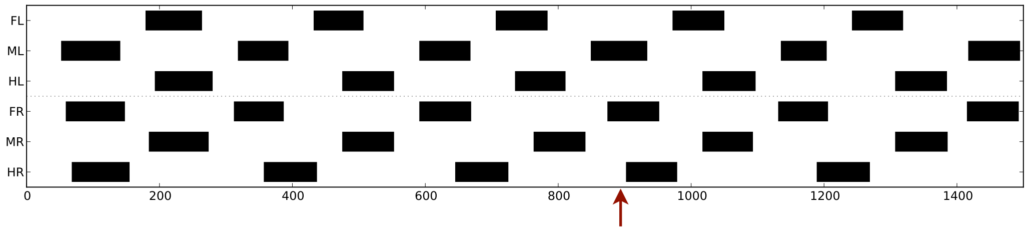

Leg position over time (shown is leg movement from posterior (negative y values) to anterior (positive y values) over time. The arrow marks the shift of the middle left leg (middle left) to the front. This is compensated during the next step by a shortened stance movement:

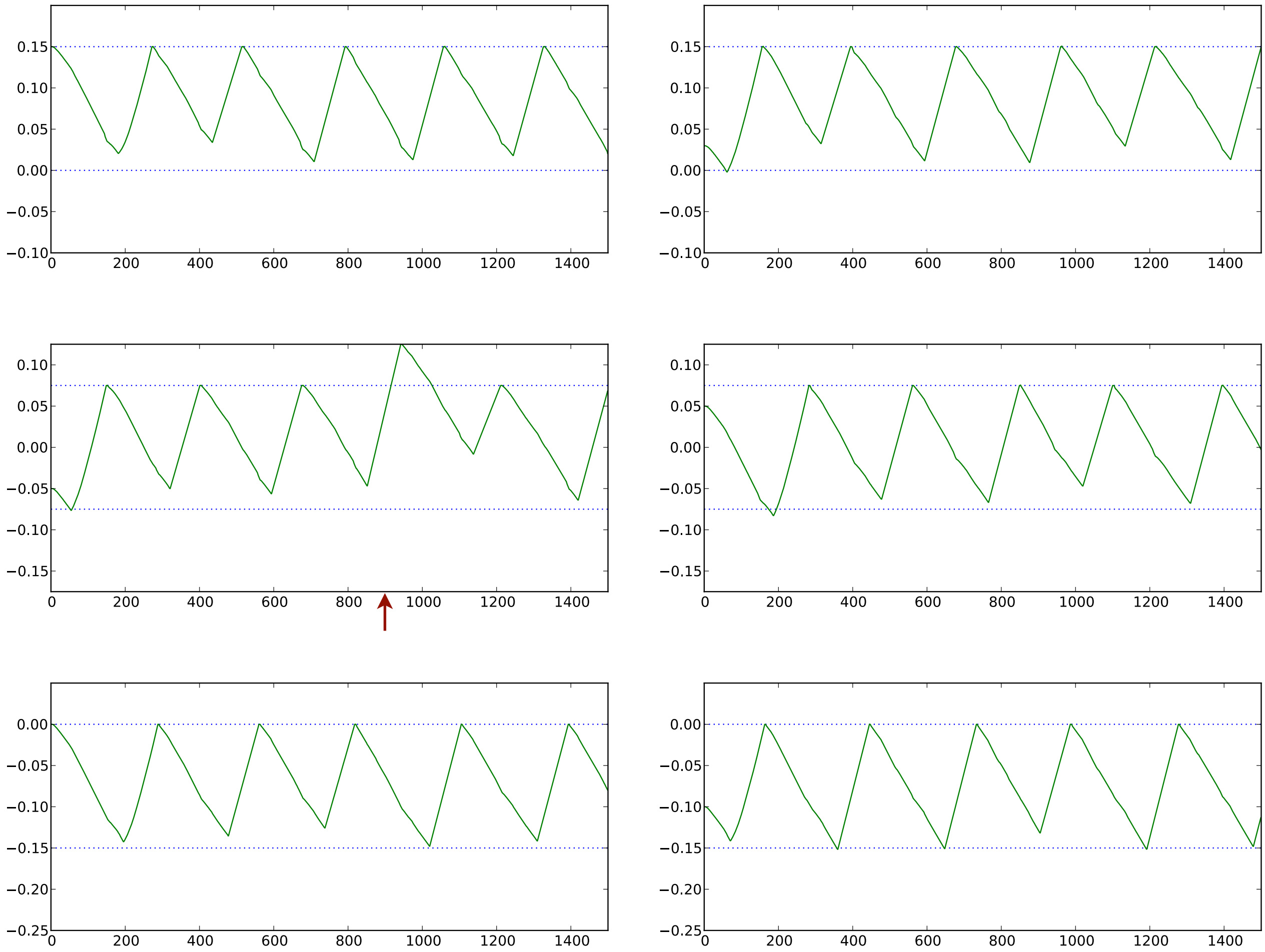

C – Hind leg disturbance

Footfall pattern for hind left leg disturbance (after six seconds):

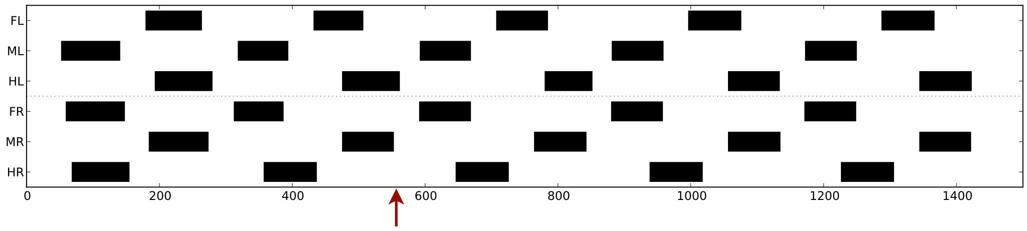

Leg position over time (shown is leg movement from posterior (negative y values) to anterior (positive y values) over time. The arrow marks the shift of the hind left leg (lower left) to the front. This is again compensated during the next step by a shortened stance movement:

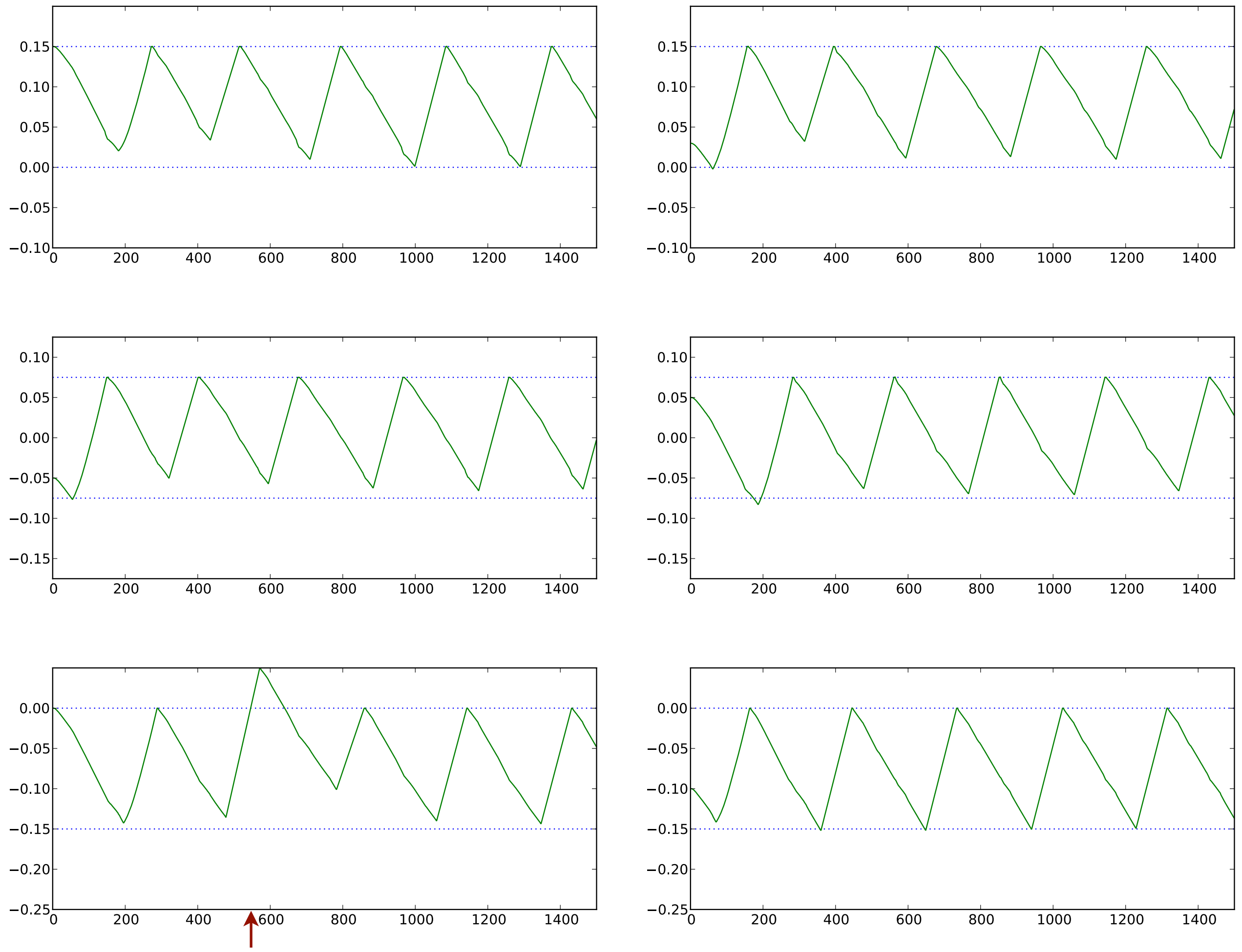

Supplemental Material 2:
Random AEP shift of two legs

For the following simulation two randomly chosen swing movements are shifted to the anterior. Shown is a disturbance of the middle right and hind left leg. The disturbance was applied after 583 iteration steps acting on the subsequent swing movement of the right middle and left hind leg.

Footfall pattern:

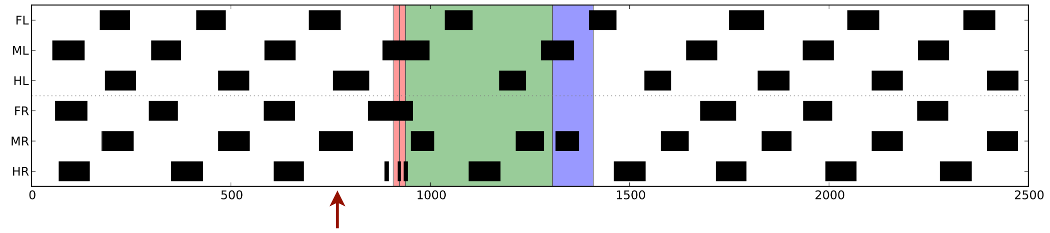

Leg position over time (shown is leg movement from posterior (negative y values) to anterior over time). In this particular situation the situation is resolved when the system makes an adjustment movement with the right middle leg. Surprisingly the system choses a movement to the front and not directed towards the back. A movement towards the back would not succeed as afterwards middle and hind right leg are in phase. The movement to the front in this case only gives the system time to settle — as a consequence the hind right leg is not forced to do an early swing movement (initiated by the contralateral hind leg), but is only switching towards a swing movement when hind left and middle right have moved further backward and providing sufficient support.

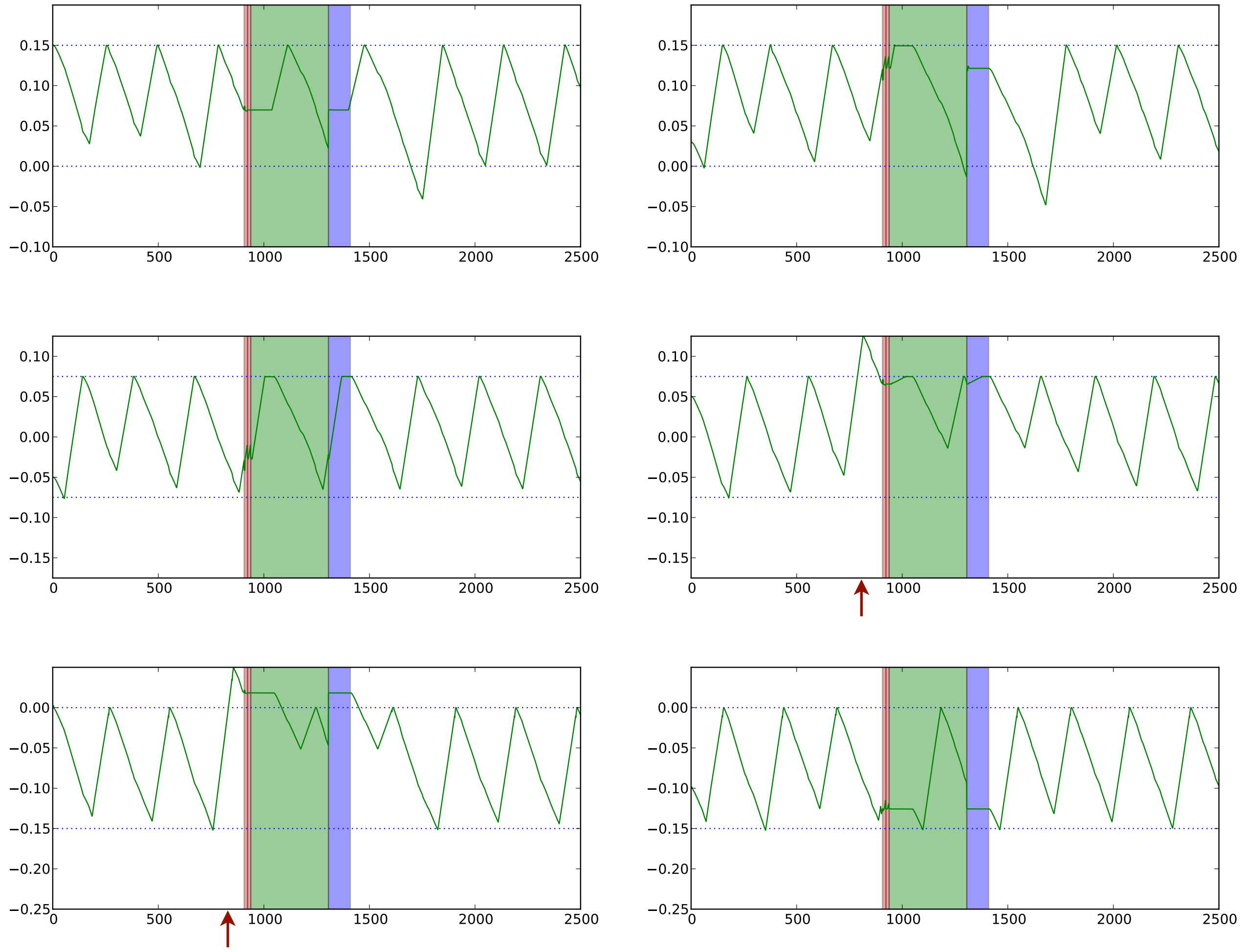

Supplement: Supplemental Data 1 — Showing example runs from additional simulations. [file DataSheet1.PDF]
